# Identification of biomarkers for abdominal aortic aneurysm in Behçet's disease via mendelian randomization and integrated bioinformatics analyses

**DOI:** 10.1111/jcmm.18398

**Published:** 2024-05-24

**Authors:** Chunjiang Liu, Huadong Wu, Kuan Li, Yongxing Chi, Zhaoying Wu, Chungen Xing

**Affiliations:** ^1^ Department of General Surgery The Second Affiliated Hospital of Soochow University Suzhou China; ^2^ Department of vascular surgery First affiliated Hospital of Huzhou University Huzhou China; ^3^ Department of General Surgery Kunshan Hospital of Traditional Chinese Medicine Kunshan China

**Keywords:** abdominal aortic aneurysm, Behçet's disease, bioinformatics analysis, diagnostic biomarker, machine learning

## Abstract

Behçet's disease (BD) is a complex autoimmune disorder impacting several organ systems. Although the involvement of abdominal aortic aneurysm (AAA) in BD is rare, it can be associated with severe consequences. In the present study, we identified diagnostic biomarkers in patients with BD having AAA. Mendelian randomization (MR) analysis was initially used to explore the potential causal association between BD and AAA. The Limma package, WGCNA, PPI and machine learning algorithms were employed to identify potential diagnostic genes. A receiver operating characteristic curve (ROC) for the nomogram was constructed to ascertain the diagnostic value of AAA in patients with BD. Finally, immune cell infiltration analyses and single‐sample gene set enrichment analysis (ssGSEA) were conducted. The MR analysis indicated a suggestive association between BD and the risk of AAA (odds ratio [OR]: 1.0384, 95% confidence interval [CI]: 1.0081–1.0696, *p* = 0.0126). Three hub genes (*CD247*, *CD2* and *CCR7*) were identified using the integrated bioinformatics analyses, which were subsequently utilised to construct a nomogram (area under the curve [AUC]: 0.982, 95% CI: 0.944–1.000). Finally, the immune cell infiltration assay revealed that dysregulation immune cells were positively correlated with the three hub genes. Our MR analyses revealed a higher susceptibility of patients with BD to AAA. We used a systematic approach to identify three potential hub genes (*CD247*, *CD2* and *CCR7*) and developed a nomogram to assist in the diagnosis of AAA among patients with BD. In addition, immune cell infiltration analysis indicated the dysregulation in immune cell proportions.

## 1 INTRODUCTION

Behçet's disease (BD) is a persistent systemic inflammatory disorder characterised by an underlying chronic vasculitis, reflecting an inflammatory process affecting the blood vessels. Vascular involvement is observed in 15% to 40% of patients with BD; among these individuals, approximately 27.5% could present with vascular lesions as their initial manifestation.^1^ Inflammation can lead to arterial aneurysms, thrombosis and endothelial dysfunction. A combination of BD with aneurysm involves true aneurysm, pseudoaneurysm and aortic dissection, with the abdominal aorta as the most common site of occurrence.^2^

Abdominal aortic aneurysm (AAA) is distinguished by the infiltration of immune cells, heightened proteolytic activity and persistent degradation of extracellular matrix constituents, including collagen, elastin, fibronectin and laminin, thus expanding the aortic wall.^3^ Although the involvement of AAA in BD is rare, it can be associated with severe consequences. AAA is characterised by progressive aortic dilation that may result in a potentially lethal rupture. Fortunately, endovascular therapy with proper medicines for the treatment of AAA in patients with BD has yielded promising results with low morbidity and mortality.^4^, ^5^ Therefore, early diagnosis and treatment of AAA in patients with BD are of utmost importance to prevent the rupture of AAA. Biomarkers can facilitate timely detection and medical intervention for conditions such as BD and asymptomatic AAA, which typically lack discernible clinical manifestations.

Microarray‐based gene expression profiling has been recently and widely applied to biomedical and clinical research as a prominent biomarker tool.^6^ Multiple studies have successfully identified biomarkers associated with BD and AAA. For instance, CLEC12A, IFI27 and CLC are considered potential and valuable biomarkers for the diagnosis of BD.^7^ A study reported the association of miR‐24 and CHI3L1 as biological markers with AAA.^8^ Another study reported the involvement of *MEDAG* and *SERPINE1* genes in the pathogenesis of AAA.^9^ The aetiology of BD remains incompletely elucidated. However, infection‐related, genetic, epigenetic and immunological factors are known to collectively contribute to its progression.^10^ The immune‐mediated infiltration and subsequent destruction of the aortic wall have been implicated in the development of AAA,^11^ with an impaired inflammatory response playing a significant role in inducing AAA in patients with BD.^12^ Therefore, biomarkers linked to immune filtration can be useful in predicting the susceptibility of patients with BD to AAA and aid in its treatment. Nevertheless, the literature on the genetic mechanism underlying BD‐induced AAA is little and warrants further investigation.

Mendelian randomization (MR) analysis, a promising epidemiological method, has been proposed to accurately evaluate the potential causal relationships. Moreover, MR analysis effectively mitigates potential confounding factors and reverses causality by leveraging the random allocation principle of alleles and employing instrumental variables (IVs) as genetic variants.^13^ In the present study, we used summary‐level statistics from previous genome‐wide association studies (GWASs) to conduct an MR analysis, facilitating a more feasible exploration of potential causal relationships between BD and AAA.

Significant advances have occurred in the fields of bioinformatics and machine learning over the past decade.^14^, ^15^, ^16^, ^17^ These advancements have facilitated the investigation of underlying mechanisms and the identification of potential biomarkers.^18^, ^19^, ^20^, ^21^ For instance, Limma analysis and weighted gene co‐expression network analysis (WGCNA) were employed to identify differentially expressed genes (DEGs) in BD and AAA, as well as specific module genes significantly associated with both conditions. Previous studies have employed this method to identify shared risk genes associated with different phenotypes of disease.^22^ A combination of protein–protein interaction (PPI) network analysis, machine learning algorithms and evaluation of nomogram results was used to ascertain significant biomarkers associated with AAA and BD.

To the best of our current understanding, research investigating the genetic mechanism underlying BD‐induced AAA is lacking. In the current study, we used integrated bioinformatics and machine learning techniques to identify significant biomarkers associated with BD‐induced AAA.

## 2 METHODS

### 2.1 Mendelian randomization (MR) analysis

**FIGURE 1:**
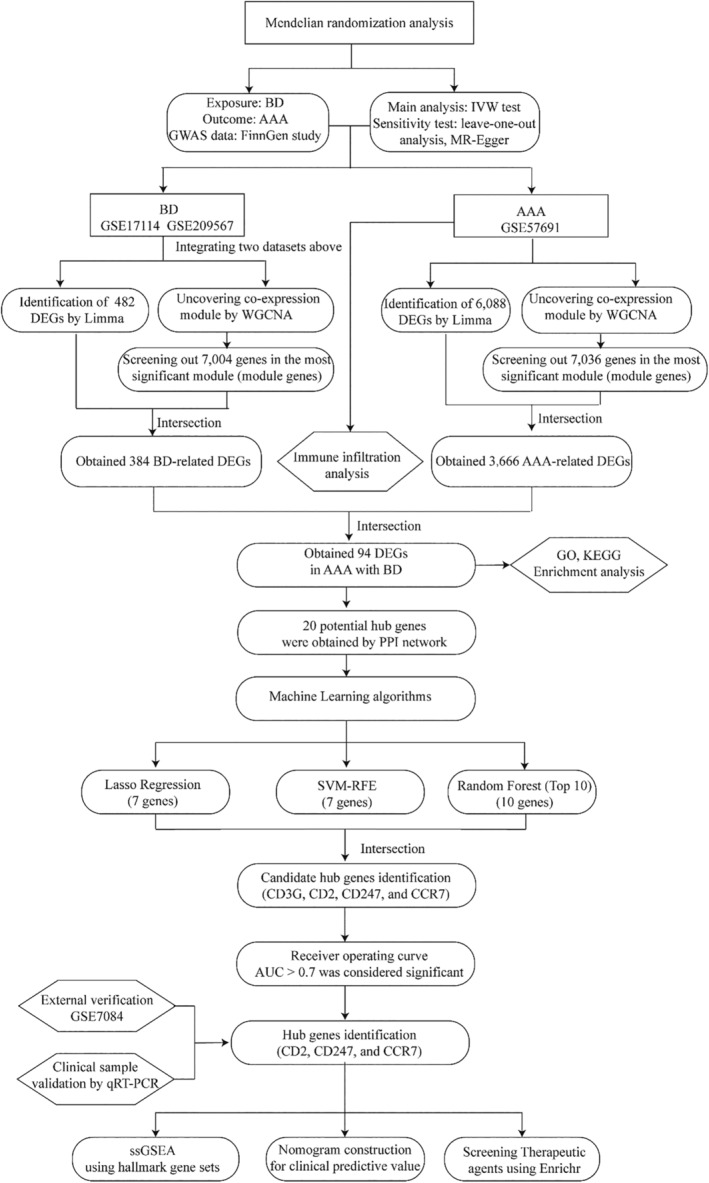
Study flowchart.

Figure 1 depicts the study flowchart. We used two‐sample MR analysis to examine the causal link between BD and the likelihood of AAA.^23^ The GWAS summary statistics for BD and AAA were acquired from the 9th FinnGen study. BD was treated as the exposure variable and AAA as the outcome measure. Three crucial assumptions must be met to conduct an MR study: Firstly, the selected single nucleotide polymorphisms (SNPs) should exhibit a significant correlation with the exposure (BD). Secondly, the SNPs should be independent of the potential confounding factors. Thirdly, the SNPs should be specifically related to the risk of the outcome (AAA) through BD. Genetic variants with genome‐wide significant (*p* < 5 × 10^−5^) association with BD were selected as instrumental variables. The causal effect of BD on the risk of AAA was examined in an inverse variance‐weighted (IVW) meta‐analysis using the Wald ratio estimates.^24^ The MR–Egger test was analysed to detect potential pleiotropy. A *p*‐value exceeding 0.05 for the MR–Egger intercept was indicative of the absence of horizontal pleiotropy. The stability was evaluated using leave‐one‐out sensitivity analyses, wherein a single SNP was excluded in each iteration. A two‐sided *p*‐value below 0.05 was considered significant. Statistical analyses were conducted using the “two‐sample‐MR,” “MR‐PRESSO,” and “mr. raps” packages.^25^

### 2.2 Microarray data

Four microarray datasets (GSE17114, GSE209567,^7^ GSE57691 and GSE7084^26^) were downloaded them from the NCBI Gene Expression Omnibus (GEO) database.^27^ The search strategy used the terms “Behçet's disease” or “abdominal aortic aneurysm” in conjunction with “*Homo sapiens*” AND “expression profiling by array.” Datasets GSE17114 and GSE209567 included gene expression data on patients with BD and normal controls. Datasets GSE57691 and GSE7084 contained data on patients with AAA and controls. Table 1 presents comprehensive details on datasets, encompassing the microarray platform used, sample groups involved and their respective quantities. The datasets underwent preprocessing using the “afy” package in R, sourced from the Bioconductor project, for background calibration and normalisation. The median expression of multiple probes corresponding to the same gene was calculated. The probes were subsequently converted to gene symbols and organised into matrix files. The GSE17114 and GSE209567 datasets were merged using the R/Bioconductor inSilicoDb package. The R “surrogate variable analysis” package was used to eliminate batch effects and other undesired variations.

**TABLE 1:** Detailed information of the GEO datasets in the study.

ID	GSE series	Disease	Samples	Source types	Platform	Group
1	GSE17114	BD	15 BD patients and 14 normal controls	Peripheral blood	GPL570	Discovery cohort
2	GSE209567	BD	29 BD patients and 15 normal controls	Peripheral blood	GPL570	Discovery cohort
3	GSE57691	AAA	49 AAA patients and 10 donors	Aortic wall	GPL10558	Discovery cohort
4	GSE7084	AAA	9 AAA patients and 10 donors	Aortic wall	GPL2507 GPL570	Validation cohort

### 2.3 Differentially expressed gene (DEG) analysis using Limma

Linear models for microarray data (Limma) are a differential expression screening method that utilises generalised linear models to identify differentially expressed genes (DEGs) between different comparison and control groups. The method involves applying the lmFit function to conduct multiple linear regression on the acquired expression profile dataset. Subsequently, the eBays function is used to calculate moderated *t*‐statistics, moderated F‐statistics and log‐odds of differential expression through empirical Bayes moderation of the standard errors toward a common value. The Limma package^28^ was used to screen for DEGs between BD and the control group in the merged dataset (GSE17114 and GSE209567). Next, the same package was used to detect the DEGs between AAA and control groups in the GSE57691 dataset. The criteria for screening DEGs were a *p*‐value below 0.05 and a fold change (FC) exceeding 1.2.

### 2.4 Significant module identification using weighted gene co‐expression network analysis (WGCNA)

WGCNA has been extensively used to construct gene co‐expression networks.^29^ The method clusters genes exhibiting similar expression patterns and assesses the correlation between gene modules and specific phenotypes. We used WGCNA to ascertain significant module genes exhibiting a strong correlation with BD and AAA. To begin, the median absolute deviation (MAD) was calculated for each gene. Subsequently, 50% of genes with the lowest MAD values were excluded. Afterward, a scale‐free co‐expression network was established by applying the goodSamplesGenes function in WGCNA to filter the expression matrix of DEGs. Subsequently, the adjacency was determined using the pick‐soft‐threshold function, which uses the co‐expression similarity to derive the soft thresholding power β. In our study, we set the soft thresholding power β to 8 for BD and 10 for AAA (Figures S1 and S2). Afterward, the adjacency was transformed into a topological overlap matrix (TOM), and the gene ratio and associated dissimilarity were computed. Genes were hierarchically clustered based on their dissimilarity degree (1‐TOM) to group genes with similar expression patterns into gene modules. The minimum module size was defined as containing 100 genes. Ultimately, the identification of modules was achieved through hierarchical clustering and dynamic tree‐cutting techniques.

### 2.5 Functional enrichment analysis

Functional enrichment analysis, which encompassed GO and KEGG analyses was conducted using the clusterprofiler package in R.^30^ The GO analysis categorised functions into biological processes (BP), molecular functions (MF) and cellular components (CC), with the top 10 GO terms in each category visualised using the “ggplot2” package in R while applying the screening criteria of false discovery rate <0.05 and *p‐*value <0.05.

### 2.6 Protein–protein interaction (PPI) network construction

The potential interplay of the identified DEGs was investigated by mapping them to the PPI network. The PPI network was established using the STRING 12.0 database,^31^ with a minimum required interaction score of 0.400. DEGs that were non‐protein‐coding and lacked interactions with other DEGs were excluded from the analysis. A visualisation of the PPI network was created using the remaining DEGs. The data in “tsv” format was acquired and imported into the Cytoscape software (version 3.9.1).^32^ The importance of nodes in biological networks and the identification of central elements can be determined by measuring their network features. Subsequently, three algorithms (betweenness, closeness and degree) were selected to evaluate the topological characteristics of each node in the interaction network.^33^ They are crucial topological algorithms used to evaluate node significance within a network and determine whether a target protein serves as a fundamental basis for key targets. The DEGs underwent additional filtration using three algorithms in the CytoHubba plug‐in within Cytoscape. Each algorithm assigned a score to each DEG, resulting in the ranking of DEGs according to their scores. The top 30 DEGs, determined by each algorithm, were designated as node DEGs. The interaction of each algorithm with the node DEGs was visually depicted using a Venn diagram.

### 2.7 Machine‐learning algorithms

Machine learning techniques have successfully identified hub genes associated with various diseases. Prominent machine learning algorithms commonly employed to screen potential biomarkers include the Support Vector Machine‐Recursive Feature Elimination (SVM‐RFE), least absolute shrinkage, selection operator (Lasso) and random forest.^34^ The risk of overfitting was avoided using the least absolute shrinkage and selection operator (Lasso)^35^ regression analysis for variable filtration. The Support Vector Machine‐Recursive Feature Elimination (SVM‐RFE)^36^ algorithm is suitable for datasets with limited samples as it eliminates redundant factors, retaining only relevant variables. Moreover, random forest^37^ is advantageous in managing datasets with numerous dimensions, constructing predictive models and estimating the significance of individual variables. Consequently, the intersection of three algorithms was used to obtain DEGs, which were regarded as potential biomarkers.

### 2.8 Evaluation of receiver operating characteristic curve (ROC) and nomogram

Student's *t*‐test was used to compare candidate gene expression between AAA and control groups. A ROC curve was constructed, and the corresponding area under the curve (AUC) with a 95% confidence interval (CI) was calculated to evaluate the diagnostic performance of each gene.^38^ Furthermore, we developed a nomogram using the R package “rms.”^39^ The nomogram converts the relative expression of each gene into a score, which is subsequently aggregated to form the total score to predict the incidence of BD with AAA. In addition, a ROC curve was generated to assess the performance of the nomogram. Only AUC >0.7 was considered significant in patients with BD having AAA.

### 2.9 Peripheral blood collection, validation of the expression of hub genes and evaluation of the predictive model

To further validate the hub genes we identified, we collected peripheral blood samples from patients diagnosed with BD (*n* = 8) and BD complicated with AAA (*n* = 4) at the First Affiliated Hospital of Huzhou University between 1 August 2022 and 1 March 2024. All BD patients were diagnosed by the International Criteria for BD.^40^ All AAA patients were diagnosed by contrast‐enhanced CT. The basic clinical characteristics of all participants are provided in Table S1. Approval for the sample collection protocol was obtained from the Ethics Committee of the First Affiliated Hospital of Huzhou University (Huzhou, China). After collecting peripheral blood, the MolPure® Blood RNA Kit (19241ES50, Yeasen, Shanghai, China) was used to extract total RNA, following the provided instructions. Subsequently, the concentration of RNA was assessed using Nanodrop (Thermofisher, USA), and cDNA synthesis was performed using the Hifair® II 1st Strand cDNA Synthesis Kit (11121ES60, Yeasen, Shanghai, China). The primers used in this study are listed in Table S2. Finally, the expression levels of the hub genes were detected between the two groups. In addition, a predictive nomogram model was constructed to distinguish BD patients with or without AAA.

### 2.10 Immune infiltration analysis, single‐sample gene‐set enrichment analysis (ssGSEA) and therapeutic agents screening

The composition of infiltrating immune cells from the normalised gene expression matrix was determined using the “Cibersort” algorithm. The R package “Cibersort” was utilised to quantify the proportions of 22 distinct types of immune cells between AAA and control groups. The proportions of immune cells were visualised using bar plots, and differences in immune cell expression between the two groups were measured using boxplots. The correlation between different immune cells in the development of AAA was illustrated using a heatmap generated with the R package “corrplot.”^41^ The relationship between immune cell infiltrations and characteristic genes was examined using ssGSEA. A *p*‐value < 0.05 was considered statistically significant.

The association between potential biomarkers and hallmark gene sets was established using the ssGSEA method. Initially, a comprehensive set of 50 well‐defined biological states or processes, referred to as hallmark gene sets, was acquired from MSigDB.^42^ Subsequently, the GSVA package^43^ was used to conduct ssGSEA and determine the correlation between potential biomarkers and hallmark gene sets. A *p*‐value < 0.05 was considered statistically significant.

Finally, Enrichr (https://maayanlab.cloud/Enrichr/) was employed to screen for therapeutic agents targeting the hub genes, with a threshold of *p*‐value < 0.05 being utilised for the enrichment analysis.

### 2.11 Statistical analysis

Statistical analyses were carried out utilizing the R software (version 4.2.1), GraphPad Prism (version 9.4.0) and SPSS (version 26.0). The continuous variables between the two groups were compared using Student's *t*‐test, with statistical significance considered when the *p*‐value was below 0.05.

## 3 RESULTS

### 3.1 MR analysis of genetic susceptibility to BD and AAA

**FIGURE 2:**
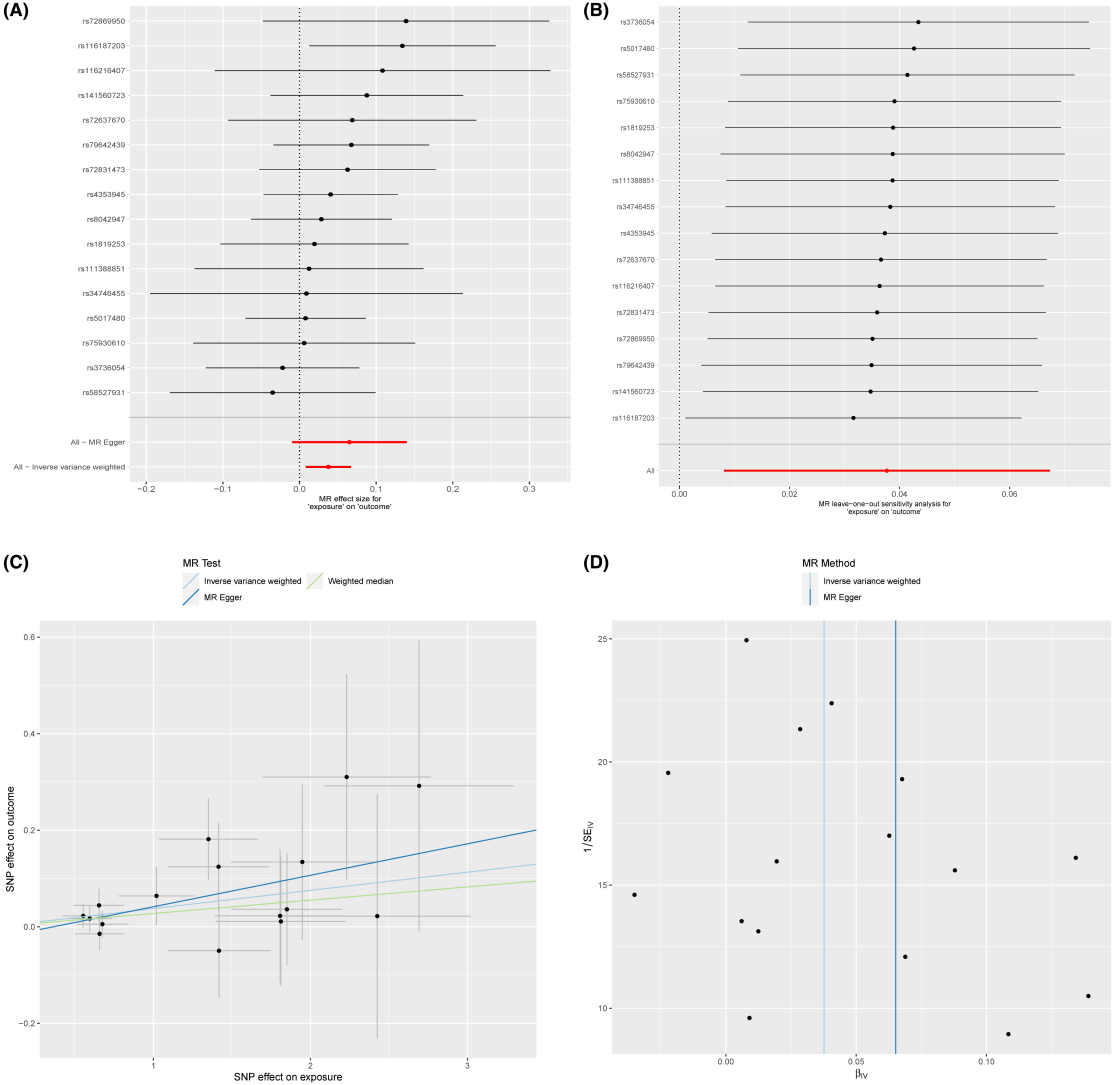
Causality of BD on AAA risk. (A) The red points illustrate the integrated estimates obtained using the IVW method with all SNPs analysed collectively. The horizontal lines indicate the corresponding 95% confidence intervals. (B) In the leave‐one‐out analysis, the IVW method was employed to assess the causal effect. This involved excluding one specific variant from the analysis. The resulting estimates are represented by black points, while the inverse‐variance weighted estimate using all SNPs is depicted by the red point. (C) The scatter plot demonstrates the estimated effects of each method of Mendelian randomization, with the slope of each line indicating the magnitude of the effect. (D) The funnel plot demonstrates the estimates with all SNPs, indicated by vertical lines.

In the MR analysis, we excluded SNPs associated with confounding factors and outcomes as well as SNPs with incompatible alleles or exhibiting palindromic patterns with intermediate allele frequencies. Subsequently, we retained 16 BD‐related SNPs that met the three crucial assumptions of the MR study for further analysis. Table S3 lists the comprehensive details of IVs for BD in MR analysis. The IVW analysis indicated a suggestive association between BD and the risk of AAA (OR: 1.0384, 95% CI: 1.0081–1.0696, *p* = 0.0126). The MR–Egger regression analysis was conducted to examine the presence of horizontal pleiotropy. The findings showed that pleiotropy is unlikely to introduce bias to the causal relationship (*p* > 0.05; Figure 2 and Table S4).

### 3.2 DEG identification via Limma in BD and AAA

**FIGURE 3:**
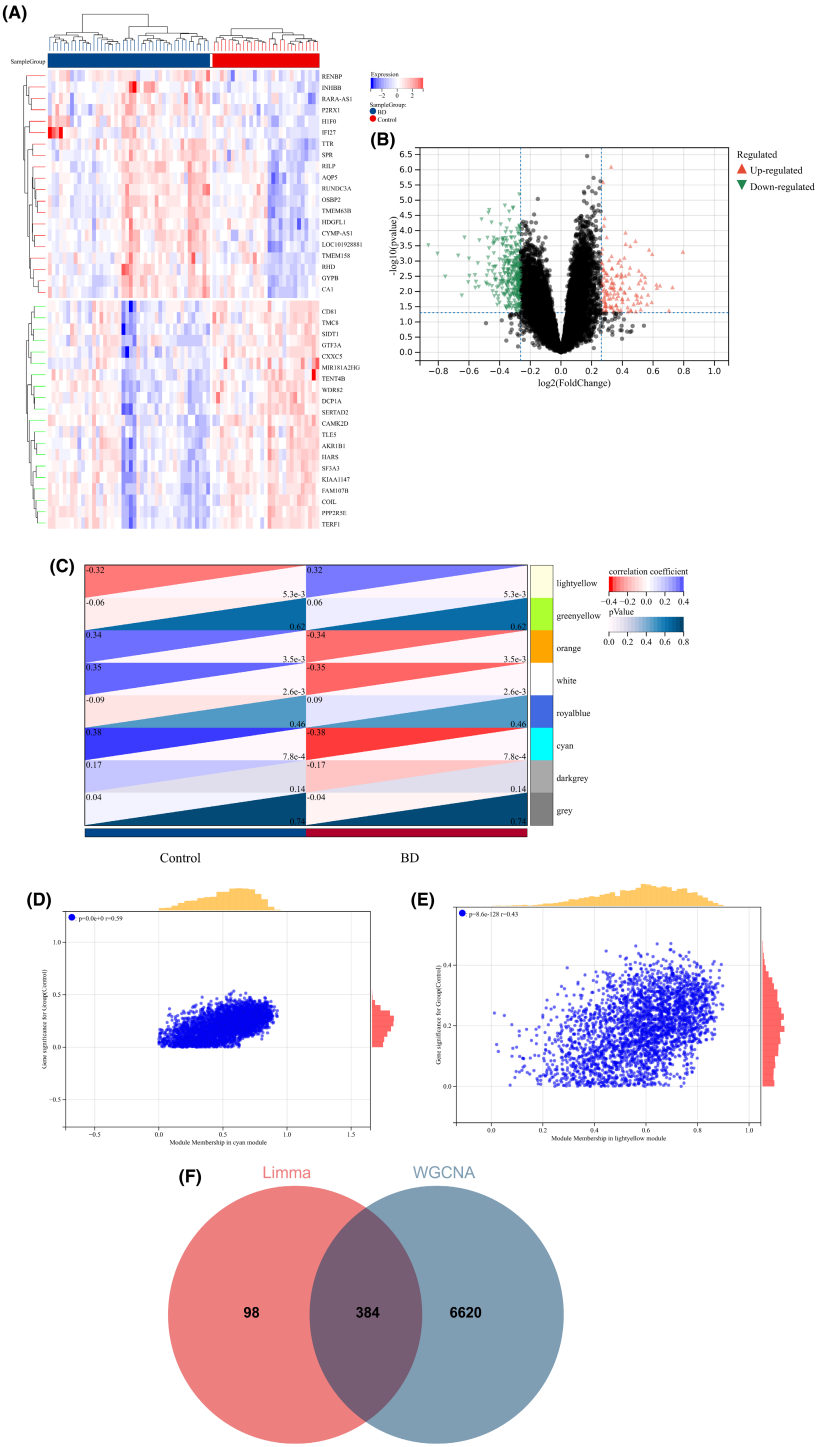
Heatmap and volcano plot of DEGs between BD and the control group. The recognition of module genes using WGCNA in BD. (A) Heatmap displaying the expression of the 20 most upregulated and downregulated DEGs. The red block indicates upregulation, reflecting increased gene expression in BD, whereas the blue block indicates downregulation, indicating decreased gene expression. (B) Volcano plot presenting all DEGs, with significantly upregulated labelled in red and downregulated in green. (C) The heatmap displays BD‐associated modules. The correlation values between each module and BD are shown in the top left corner of the heatmap, and the *p*‐values in the lower right corner signify the statistical significance of the correlation. In BD, the light yellow and cyan modules exhibit the strongest correlation. (D, E) The correlation between gene significance in BD and module membership within the cyan/light yellow module. (F) The intersection of 482 DEGs and 7004 BD‐associated module genes led to the identification of 384 BD‐related DEGs.

**FIGURE 4:**
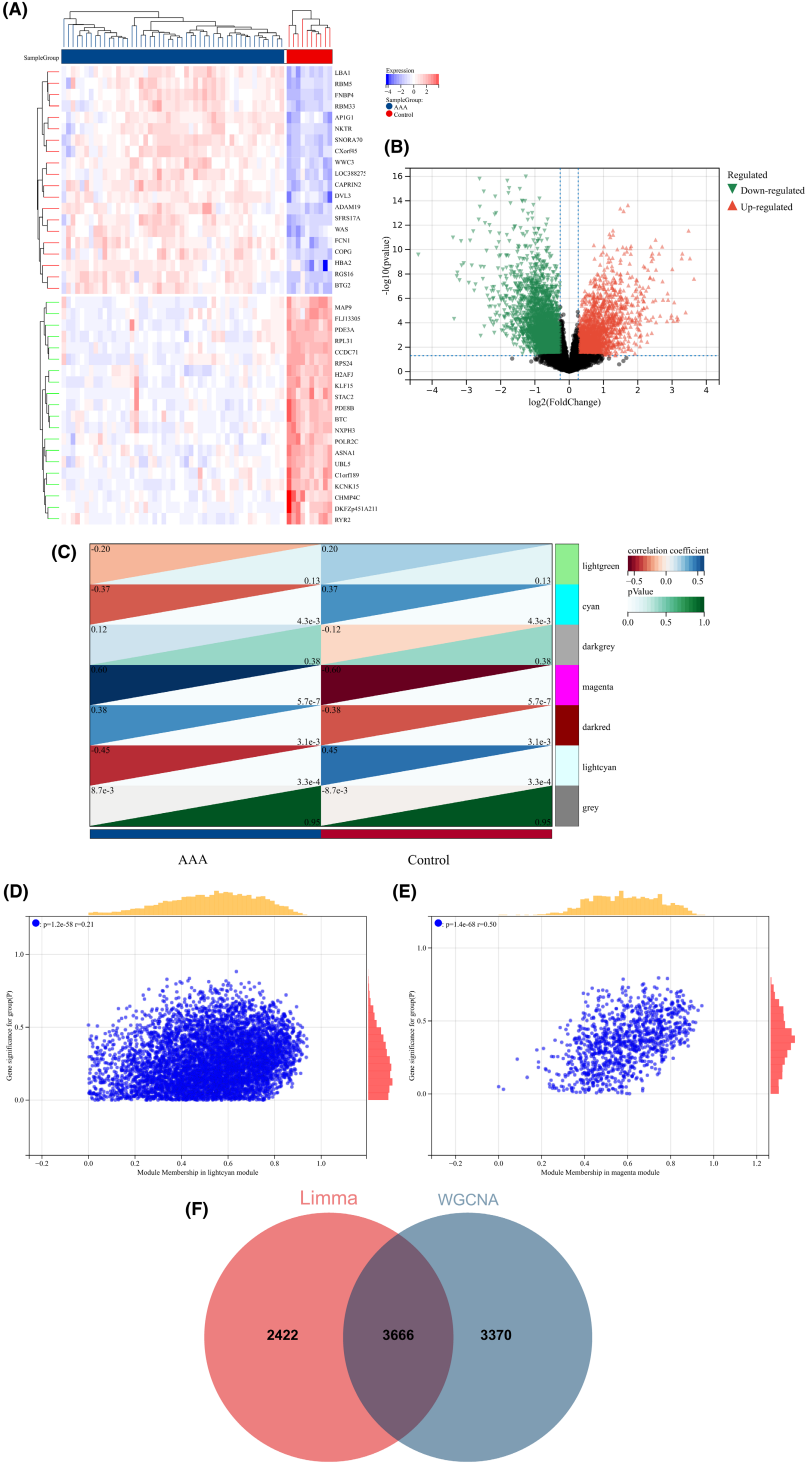
Heatmap and volcano plot of DEGs between AAA and the control group; WGCNA recognition of module genes in AAA. (A) Heatmap displaying the expression of the 20 most upregulated and downregulated DEGs. The red block indicates upregulation, reflecting increased gene expression in AAA, whereas the blue block indicates downregulation, indicating decreased gene expression. (B) Volcano plot presenting all DEGs, with significantly upregulated labelled in red and downregulated in green. (C) The heatmap displays AAA‐associated modules. The correlation values between each module and AAA are shown in the top left corner of the heatmap, and the *p*‐values in the lower right corner signify the statistical significance of the correlation. In AAA, the light cyan and magenta modules exhibit the strongest correlation. (D, E) The correlation between gene significance in AAA and module membership within the light cyan/magenta module. (F) The intersection of 6088 DEGs and 7036 module genes associated with AAA led to the identification of 3666 AAA‐related DEGs.

A total of 482 DEGs were identified by comparing the BD and control groups. Among these, 134 genes displayed upregulation, whereas 348 genes were downregulated. Similarly, a comparison of AAA and control groups revealed 6088 DEGs, of which 2363 genes were upregulated and 3725 genes were downregulated. A heatmap was used to display the 20 most significant DEGs exhibiting either upregulation or downregulation. In addition, a volcano plot was used to represent all DEGs (BD: Figure 3A,B; AAA: Figure 4A,B).

### 3.3 Identification of significant module genes in BD and AAA via WGCNA

Next, WGCNA was used to identify the significant module genes associated with BD and AAA. The grey module did not successfully cluster the genes commonly considered irrelevant or uninformative (i.e., the “junk module”). The light yellow (*r* = 0.32, *p* = 5.3 × 10^−3^) module and cyan (*r* = −0.38, *p* = 7.8 × 10^−4^) module displayed the highest correlation with BD (Figure 3C). Figure 3D,E depict the relationship between module membership and gene significance in the cyan/light yellow module of BD. The light cyan module demonstrated the highest positive correlation with AAA (*r* = 0.45, *p* = 3.3 × 10^−4^), whereas the magenta module displayed the strongest negative correlation (*r* = −0.60, *p* = 5.7 × 10^−7^) (Figure 4C). Figure 4D,E depict the relationship between module membership and gene significance in the light cyan/magenta module of AAA. Consequently, 7004 genes were identified in the BD group, whereas 7036 genes were identified in the AAA group. Subsequently, the intersection of 482 DEGs and 7004 BD‐associated module genes led to the identification of 384 BD‐related DEGs (Figure 3F). Similarly, the intersection of 6088 DEGs and 7036 module genes associated with AAA led to the identification of 3666 AAA‐related DEGs (Figure 4F). Figures S1 and S2 display the soft threshold selection and gene cluster tree, respectively.

### 3.4 Functional enrichment analysis of BD‐related DEGs in AAA

**FIGURE 5:**
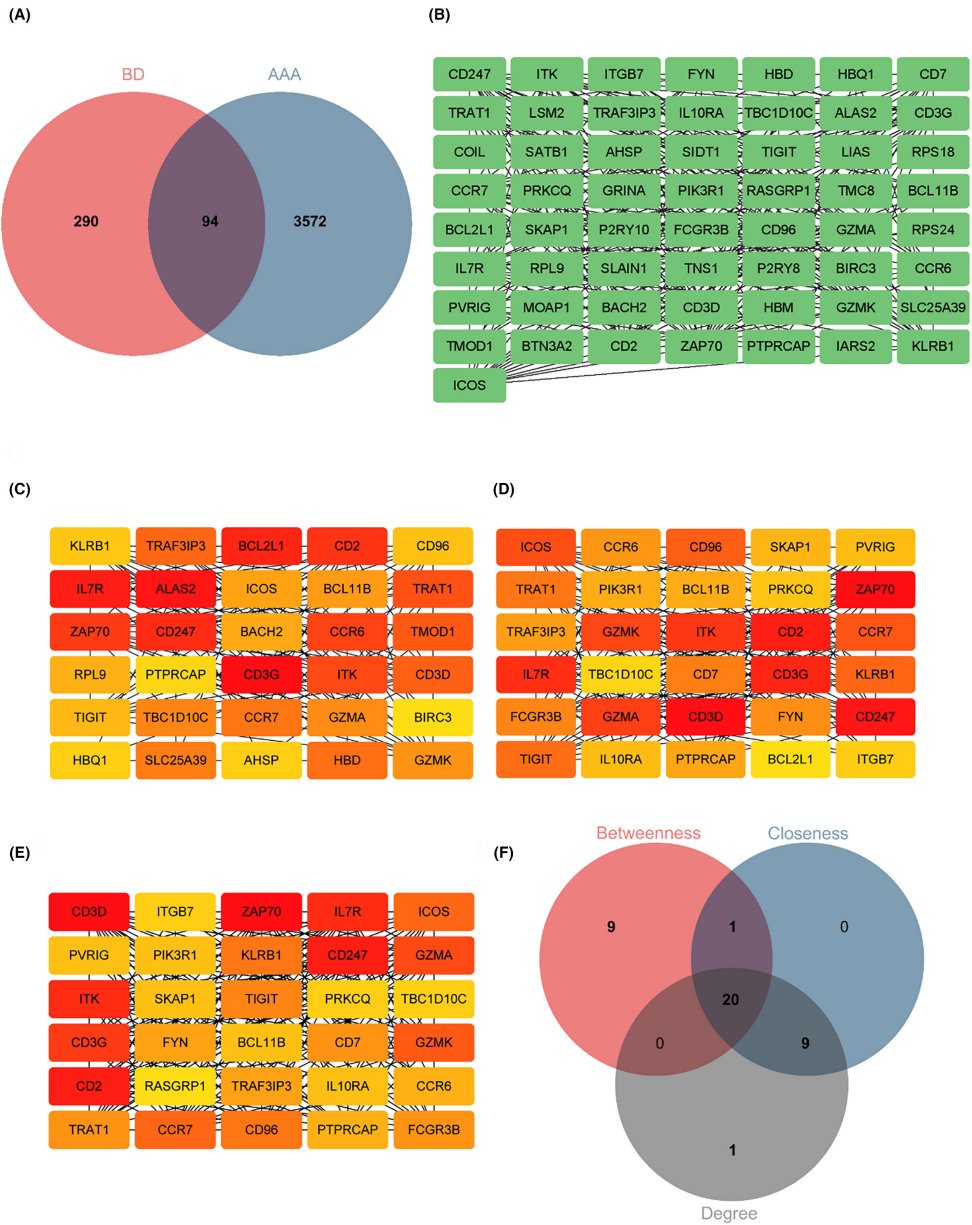
Construction of the PPI network and selection of hub genes. (A) Venn plot illustrating the overlap of DEGs between BD and AAA, resulting in 94 BD‐related DEGs in AAA. (B) The complete PPI network of 57 BD‐related DEGs in AAA was represented using STRING, excluding 37 DEGs due to a lack of interaction. (C–E) Within the CytoHubba plug‐in of Cytoscape, three distinct algorithms were utilised to select the top 30 DEGs. Panels B–D represent the application of betweenness, closeness and degree algorithms, with darker colours indicating greater significance. (F) By considering the intersection of genes identified by all three algorithms, a total of 20 DEGs were chosen.

The identification of 94 BD‐related DEGs in AAA was achieved by intersecting 384 DEGs associated with BD and 3666 DEGs associated with AAA (Figure 5A). Ninety‐four DEGs for BP displayed significant enrichment in GO terms such as “immune system process,” “immune response,” and “T cell activation.” In addition, CC was enriched with terms such as “receptor complex,” “plasma membrane receptor complex,” and “plasma membrane protein complex.” The MF of DEGs displayed significant associations with terms such as “protein kinase binding,” “protein tyrosine kinase binding,” and “non‐membrane spanning protein tyrosine kinase activity” (Figure S3 A–C and Table S5). The functional pathway analysis of 94 DEGs, depicted in Figure S3D and Table S3 revealed significant enrichment in pathways such as “T cell receptor signaling pathway,” “PD‐L1 expression and PD‐1 checkpoint pathway in cancer,” and “primary immunodeficiency.”

### 3.5 PPI network construction and potential hub gene selection

A preliminary PPI network was constructed using 94 DEGs to identify hub BD‐ and AAA‐associated DEGs. Interaction with others (Figure 5B) retained 57 DEGs, whereas 37 DEGs were excluded due to non‐interaction. Furthermore, the CytoHubba plug‐in was used along with three distinct algorithms (degree, betweenness and closeness) to identify intersecting DEGs. Figure 5C–E illustrates the top 30 node genes determined using the betweenness, closeness and degree algorithms. Figure 5F presents the overlap of 30 genes identified using three algorithms, among which 20 genes were identified as potential hub genes. Table S6 provides detailed information on these identified 20 genes.

### 3.6 Selection of candidate hub genes using machine learning techniques

**FIGURE 6:**
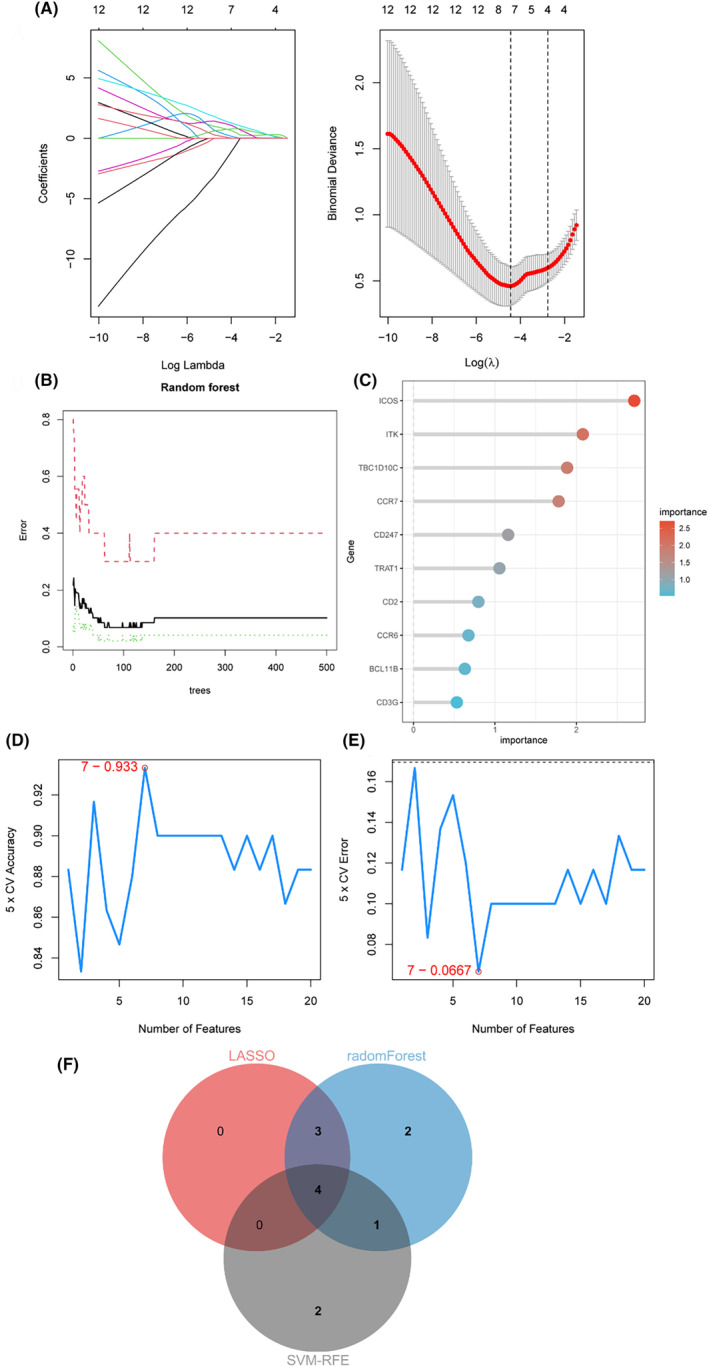
Identification of candidate diagnostic biomarkers through machine learning algorithms. (A) The Lasso regression algorithm pinpointed the curve's lowest point, identifying seven genes as highly accurate biomarkers for diagnosing BD with AAA. (B, C) The random forest algorithm was utilised to evaluate the error in AAA, resulting in the ranking of the top 10 genes based on their importance scores. (D, E) The SVM‐RFE algorithm selected 7 genes with the lowest error and highest accuracy. (F) Four candidate hub genes (*CD3G*, *CD2*, *CD247* and *CCR7*) were selected based on their intersection across the three algorithms.

We next identified seven potential hub genes that served as optimal biomarkers for diagnosing AAA in patients with BD by applying the Lasso regression algorithm. These candidate hub genes corresponded to the minimum point on the curve. Figure 6A illustrates the results of the Lasso regression. The significance of DEGs was determined using the random forest approach. The error in AAA was detected using the random forest algorithm, as shown in Figure 6B. Figure 6C displays a compilation of the 10 most significant DEGs. The SVM–RFE analysis identified the top seven DEGs, demonstrating the lowest error and highest accuracy in diagnosing AAA with BD, as depicted in Figure 6D,E. Consequently, four candidate hub genes (*CD3G*, *CD2*, *CD247* and *CCR7*) were selected based on the intersection of genes identified by the three algorithms, as shown in Figure 6F.

### 3.7 Diagnostic value evaluation and nomogram construction

**FIGURE 7:**
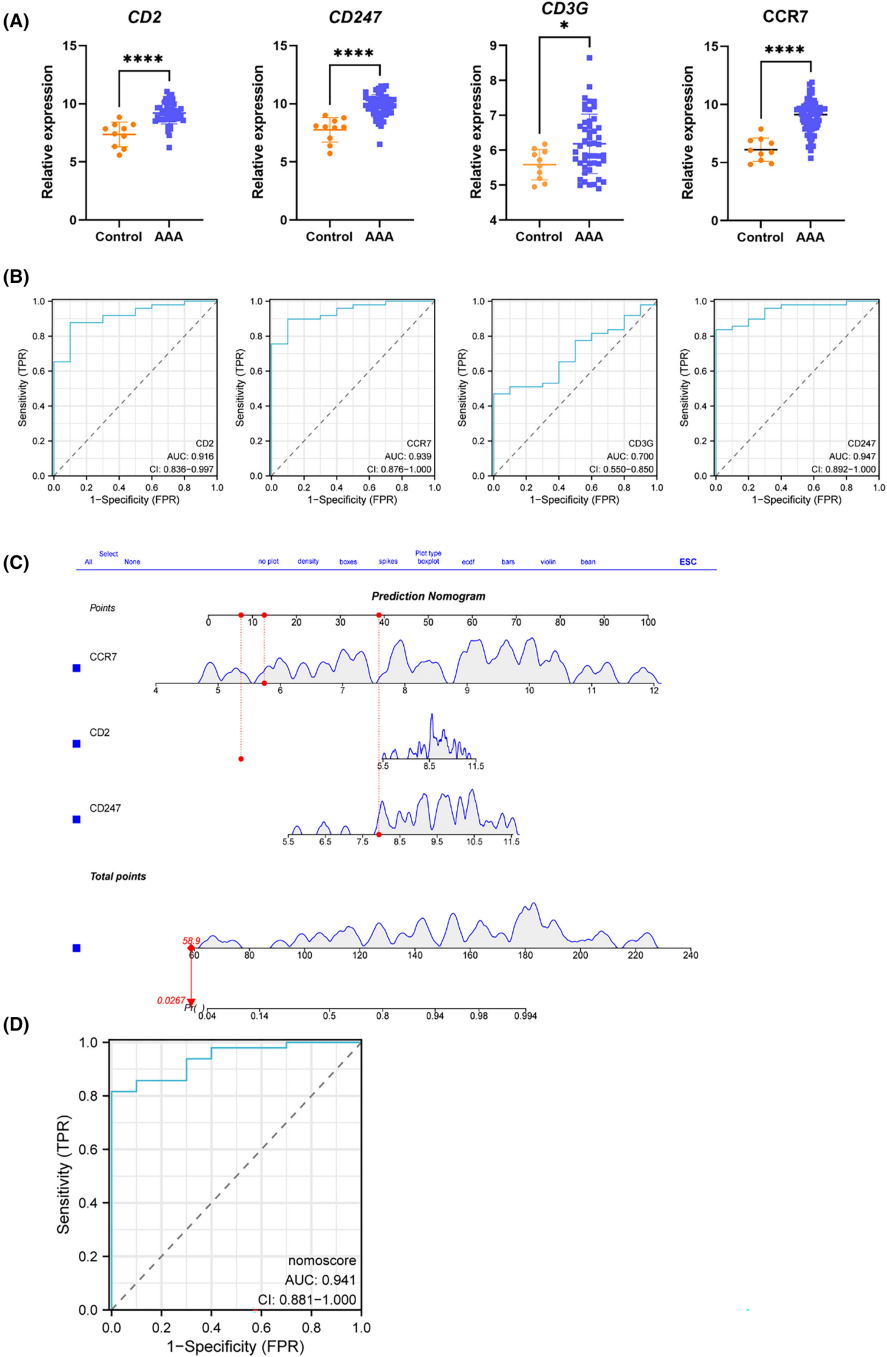
Evaluation of ROC and nomogram construction. (A) There is differential expression observed in four genes when comparing the AAA and control groups. *, *p* < 0.05; ****, *p* < 0.0001. (B) The ROC curve was used to evaluate the diagnostic efficacy of the three genes in identifying AAA with BD, showing the AUC and 95% CI in each panel. Three genes (*CD2*, *CCR7* and *CD247*) exhibit satisfactory diagnostic performance. (C) A diagnostic nomogram was constructed using the three genes to assist in the diagnosis of AAA in patients with BD. (D) ROC curve for the nomogram in AAA in patients with BD.

Compared with the control group, AAA exhibited upregulated four candidate hub genes, as shown in Figure 7A. Figure 7B presents the AUC and 95% CIs for each gene: *CD2* (AUC: 0.916, 95% CI: 0.836–0.997), *CCR7* (AUC: 0.939, 95% CI: 0.876–1.000), *CD3G* (AUC: 0.700, 95% CI: 0.550–0.850) and *CD247* (AUC: 0.947, 95% CI: 0.892–1.000). The ROC curve analyses revealed three genes (*CD2*, *CCR7* and *CD247*) that exhibited satisfactory diagnostic performance. Finally, the nomogram, as depicted in Figure 7C, yielded an AUC value of 0.941 (95% CI: 0.881–1.000), indicating a significant clinical diagnostic value, as demonstrated in Figure 7D. To further validate its diagnostic potential, the validation dataset GSE7084 was used to perform the ROC curve analysis. Compared with the control group, AAA exhibited the upregulation of the three hub genes, as shown in Figure S4A. The analysis of the nomogram in the validation dataset showed an AUC of 1.000, confirming its substantial clinical diagnostic value, as illustrated in Figure S4B,C.

### 3.8 Validation of the expression pattern of three hub genes and evaluation of the predictive value of the nomogram model

To further confirm the accuracy of the above‐integrated bioinformatics analysis, we first examined the expression pattern of the three hub genes in the recruited patients from our external cohort. All three DEGs showed upregulated expression in BD complicated with AAA compared with the BD groups. Furthermore, a predictive nomogram model was constructed to distinguish BD patients with or without AAA. The nomogram, as depicted in Figure S5, yielded an AUC value of 1, confirming its substantial clinical value in predicting the possibility of AAA in BD patients.

### 3.9 Immune cell infiltration analysis, ssGSEA and therapeutic agent screening

A bar plot, presented in Figure S6A, was used to illustrate the percentage distribution of 22 distinct immune cell types in each sample after applying the CIBERSORT algorithm. The boxplot analysis revealed a higher prevalence of B naive cells and CD4 memory‐activated T cells in AAA compared with the control group. Conversely, the proportion of M2 macrophages displayed a lower prevalence in AAA (Figure S6B). The correlation analysis demonstrated that follicular helper T cells were significantly and positively correlated with naive B cells (*r* = 0.62), whereas regulatory T cells demonstrated the highest negative correlation with CD4 memory resting T cells (*r* = −0.47), as depicted in Figure S6C. In conclusion, a mechanism involving regulating the macrophages could be a promising approach for treating AAA. Furthermore, immune cell infiltration analysis revealed a certain correlation with all three hub DEGs, as illustrated in Figure S6D.

The ssGSEA analysis revealed significant positive associations between all three hub DEGs and different biological processes, including “IL2‐STAT5 signalling pathway,” “inflammatory response,” “IL6–JAK–STAT3 signalling pathway,” “epithelial–mesenchymal transition,” and “angiogenesis,” as shown in Figure S7. Conversely, the three DEGs displayed negative correlations with the biological processes of “myogenesis,” “NOTCH signalling,” and “cholesterol homeostasis.” These biological processes could be intricately associated with the onset and progression of AAA in individuals with BD. In addition, a PPI network was constructed using 20 genes identified by three algorithms (degree, betweenness and closeness). We discovered that the three hub genes interacted through intermediate molecules (Figure S8).

The Enrichr database was utilised to screen therapeutic agents targeting the three hub genes. The predicted results indicated that methotrexate, alpha‐d‐mannose, vitinoin, ivermectin and tacrolimus monohydrate could be potential effective agents for targeting the three hub genes associated with BD‐induced AAA (Table S7).

## 4 DISCUSSION

The condition of patients with BD is complicated by abdominal aortic involvement, which can rarely exhibit risk factors for atherosclerosis. This generally occurs at a younger age. The diagnosis of patients is frequently delayed due to the asymptomatic nature of most cases. AAA represents a progressive dilation of the aorta that can ultimately result in a potentially fatal rupture. It is highly crucial to prevent, early detect and treat AAA due to the potential risk of abdominal aortic rupture in patients with BD.^44^

To our knowledge, this study represents the pioneering attempt to study the application of MR and bioinformatics analyses to investigate these two diseases. MR uses genetic variants associated with a specific biological mediator to evaluate causal relationships. It has been widely applied to investigate causal associations among different biological or medical factors. However, MR has not yet been used to investigate the causal connection between BD and AAA.

Reliable biomarkers hold paramount importance in contemporary medicine. In addition, the implementation of bioinformatics and machine learning approaches has greatly facilitated the investigation of underlying mechanisms and the identification of potential biomarkers. Therefore, these approaches can accurately identify disease‐related biomarkers, facilitate the investigation of disease occurrence and progression and enable the exploration of the underlying pathogenic mechanisms. The Limma package in R provides a comprehensive solution for analysing gene expression data. Thus, it is a widely used tool for differential gene expression analysis of microarray data. WGCNA is a systematic biological technique that analyzes gene association patterns across different samples, allowing to calculate the correlation between gene modules and phenotypes based on phenotypic information. This approach successfully identifies potential biomarkers.^45^ We utilised the STRING database to generate a protein–protein interaction biochemical network to explore the interplay among differentially expressed genes (DEGs). Subsequently, three algorithms (degree, betweenness and closeness) were used to identify central elements and hub genes based on network centrality and connectivity. A previous study has demonstrated that machine learning methods, as flexible prediction algorithms, exhibit higher accuracy compared to conventional regression.^46^, ^47^ Furthermore, stacking ensemble learning algorithms have demonstrated better performance than individual machine learning models in identifying risk factors for diseases.^48^ Thus, we employed prominent machine learning algorithms, including SVM‐RFE, Lasso and random forest, to screen potential biomarkers. The diagnostic performance of the identified genes or models was evaluated using ROC analysis and nomogram construction, assessing sensitivity, specificity and the area under the curve (AUC). This study stands out for its integration of multiple analytical methods, providing a comprehensive and advanced analysis pipeline for identifying potential key biomarkers, developing predictive models and exploring potential mechanisms for BD‐associated AAA.

Because the dataset used comprised peripheral blood samples from individuals diagnosed with BD, the assessment of hub gene expression in the peripheral blood of these patients offers valuable information to estimate the likelihood of AAA incidence in this specific population. Thus, this is a practical and efficacious clinical method. The nomogram allowed us to calculate the cumulative score of each gene and the total score. Consequently, our nomogram demonstrated substantial potential for use in clinical settings as it facilitates the identification and early intervention of patients with BD with elevated total scores, thereby improving the prognosis of this specific patient population.

Contrary to conventional observational studies, the MR analysis effectively mitigates the influence of confounding variables and reverse causation on outcomes.^49^ The MR analysis indicated a strong association between BD and the risk of AAA (OR: 1.0384, 95% CI: 1.0081–1.0696, *p* = 0.0126). The application of integrated bioinformatics, machine learning techniques and ROC evaluation successfully identified three biomarkers, namely, CD2, CD247 and CCR7. In addition, we developed a nomogram and assessed its diagnostic efficacy for AAA in patients with BD. We conducted an external validation employing an additional dataset (GSE7084), revealing noteworthy correlations between the three key genes and AAA.

CD2 belongs to the transmembrane immunoglobulin superfamily. The CD2 protein acts as a costimulatory receptor located on the surfaces of T and natural killer (NK) cells, initiating an adaptive immune response through interaction with LFA‐3/CD58 on antigen‐presenting cells (APCs).^50^ Several studies have implicated CD2 in immune responses and inflammatory disorders.^51^, ^52^ For instance, Pawlowski et al.^53^ demonstrated that the absence of CD2 can alleviate the intestinal inflammatory damage caused by *Toxoplasma gondii* infection. Similarly, Inomata et al. used monoclonal antibodies targeting CD2 molecules in mice and demonstrated that these inhibited myocardial cell injury by reducing T‐cell infiltration. Although the specific pathological mechanism contributing to the effect of CD2 on AAA is not well understood, numerous CD2^+^ T cells were detected in the cysts of patients with AAA.^54^ We detected upregulated expression of CD2 in patients with AAA, suggesting the crucial role of immune dysfunction and inflammation in the development of AAA.

CD247 encodes the T‐cell receptor (TCR) zeta, which is a critical component for assembling the TCR–CD3 complex.^55^ In humans, CD247 has been linked to several autoimmune diseases, such as rheumatoid arthritis^56^ and systemic lupus erythematosus (SLE).^57^ For example, Rudemiller et al.^58^ reported a positive correlation between CD247 and blood pressure in rats fed a high‐salt diet. In addition, knockout of *CD247* reduced the hypertension levels by decreasing immune cell infiltration into the kidneys, potentially serving as a therapeutic target to delay the progression of AAA.

CCR7, a G protein‐coupled receptor, is known to be targeted by two specific ligands, namely the C‐C motif chemokine ligand 19 (CCL19) and CCL21.^59^ Similar to other chemokine receptors, CCR7 directs the immune cells towards lymphoid organs by recognising its specific ligands. This process is essential for the initiation and maintenance of adaptive immunity.^60^ Recent research has reported a connection between CCR7, CCL19/CCL21 and several autoimmune and inflammatory diseases.^61^ For instance, Katrien et al.^62^ reported that elevated CCL21 levels in patients with rheumatoid arthritis (RA) induced the migration of CCR7^+^ monocyte macrophages into affected joints, thereby promoting the polarisation of Th17 cells and perpetuating bone erosion and vascularization. In atherosclerosis, the absence of CCR7 not only impedes the infiltration of inflammatory cells into the vascular wall but also retards the progression of atherosclerotic plaques.^63^, ^64^ We demonstrated an upregulation of CCR7 expression in individuals with AAA and BD, suggesting a potentially crucial role of CCR7 in the development of AAA in patients with BD.

Given its pathogenesis and symptomatology, BD occupies a unique position between autoimmune and autoinflammatory diseases. It is characterised by vasculitis and endothelial damage.^65^ AAA is characterised by the presence of immune cell infiltration, increased proteolytic activity and ongoing degradation of extracellular matrix components such as collagen, elastin, fibronectin and laminin. These processes ultimately expand the aortic wall. The aetiology of AAA attributed to BD remains elusive. However, the predominant hypothesis suggests that genetically predisposed individuals could experience an autoimmune response triggered by exposure to environmental factors or an autoantigen, such as a heat shock protein, resulting in the development of vasculitis. Inflammation can lead to arterial aneurysms, thrombosis and endothelial dysfunction.^66^ The GO analysis reported that the DEGs were predominantly linked to immune regulation, encompassing aspects such as immune system functioning, immune response and activation of T cells. Additionally, we analysed immune infiltration in AAA. The findings revealed an increased presence of B naive cells and CD4 memory‐activated T cells in individuals diagnosed with AAA when compared to the control group, accompanied by a lower proportion of M2 macrophages in AAA. A previous study reported the significant role of M2 macrophages in resolving the inflammatory phase, promoting tissue remodelling and ultimately inhibiting the development of AAA.^67^ Conversely, an elevated proportion of B naive cells and CD4 memory‐activated T cells could contribute to the development and rupture of AAA.^68^, ^69^ These studies are consistent with our findings.

CD2 plays a critical role in T cell activation and adhesion. BD is associated with dysregulated immune responses and increased T‐cell activation. A significant increase in peripheral blood NK cells (CD2^+^) was observed in patients with BD.^70^ Similarly, numerous CD2^+^ T cells were detected in the cysts of patients with AAA.^54^ CD247, as a component of the T cell receptor complex, regulates signal transduction in T cells.^71^ Research has demonstrated a strong association between CD2 and CD247 with immune infiltration and their association with the occurrence of AAA.^72^ High proportions of CCR7^+^ cells were observed in active patients with BD^73^ and aortic aneurysm.^74^ A bioinformatics study identified CCR7 as one of the hub genes in AAA.^75^ In this study, we examined the interplay between the three hub genes we identified and the infiltration of immune cells. We observed a positive correlation between immune‐related M1 macrophages and gamma delta T cells and the three identified hub genes (*CD2*, *CD247* and *CCR7*). The interactions between *CD247*, *CD2* and *CCR7* in the context of BD and AAA remain to be completely elucidated. Nevertheless, considering their known functions and our results, it is plausible that *CD2*, *CD247* and *CCR7* contribute to immune dysregulation and the development or progression of AAA in patients with BD. Studies pertaining to the regulation of T cells and macrophages could provide promising therapeutic strategies.

Recently, non‐coding RNAs (ncRNAs) have garnered significant attention for their functions and potential as diagnostic biomarkers in the modulation of inflammation and autoimmune diseases, including BD.^76^ Similarly, ncRNAs have been demonstrated to participate in the progression of AAA.^77^ Several important computational models, such as the graph convolutional neural (GCN) network and network distance analysis, facilitate the prediction of lncRNA–miRNA interactions.^78^, ^79^ However, the algorithms employed in this study, particularly machine learning methods, were deemed inadequate in predicting lncRNA–miRNA interactions. Future studies on lncRNA–miRNA interactions, utilizing the aforementioned important computational models, have the potential to enhance our understanding of the mechanisms underlying BD‐associated AAA and identify diagnostic biomarkers.

However, our study had several limitations. First, because both the exposure and outcome originate from the same database, a certain degree of sample duplication could be present. While the sensitivity analysis did not identify any instances of horizontal pleiotropy, it remains possible that confounding and pleiotropic factors could be present within the MR analysis. Second, irrespective of using the validation dataset (GSE7084) and clinical samples for assessing the diagnostic value, conducting additional experimental investigations is imperative to validate and explore the underlying mechanisms. The validation samples in the current study were relatively small due to the challenges associated with sample acquisition. Therefore, further verification is necessary through multicentre studies involving larger sample sizes. Third, we utilised only three commonly machine learning algorithms, and in the future, more algorithms, such as the GCN network, could be added.

## 5 CONCLUSION

Our MR analyses revealed a higher susceptibility of patients with BD to AAA. We used a systematic approach to identify three potential hub genes (*CD247*, *CD2* and *CCR7*) and developed a nomogram to assist in the diagnosis of AAA among BD patients. In addition, immune cell infiltration analysis indicated the dysregulation in immune cell proportions, suggesting a potential involvement of T cells and macrophages in AAA development.

## AUTHOR CONTRIBUTIONS

**Chunjiang Liu:** Data curation (equal); investigation (equal); project administration (equal); writing – original draft (equal). **Huadong Wu:** Data curation (equal); investigation (equal); methodology (equal); validation (equal). **Kuan Li:** Data curation (equal); investigation (equal); software (equal). **Yongxing Chi:** Software (equal). **Zhaoying Wu:** Software (equal). **Chungen Xing:** Conceptualization (lead).

## FUNDING INFORMATION

No external funding was received.

## CONFLICT OF INTEREST STATEMENT

The authors confirm that there are no conflicts of interest.

## Supporting information


Appendix S1:

## Data Availability

Data derived from public domain resourcesThe data that support the findings of this study are available inthe 9th release of the FinnGen study at https://www.finngen.fi/en/access_results and public GEO database at https://www.ncbi.nlm.nih.gov/geo. These data were derived from the following resources available in the public domain: GSE17114: https://www.ncbi.nlm.nih.gov/geo/query/acc.cgi?acc=GSE17114; GSE209567: https://www.ncbi.nlm.nih.gov/geo/query/acc.cgi?acc=GSE209567; GSE57691: https://www.ncbi.nlm.nih.gov/geo/query/acc.cgi?acc=GSE57691; GSE7084: https://www.ncbi.nlm.nih.gov/geo/query/acc.cgi?acc=GSE7084.
