# Supplementary material for: Identification of biomarkers for abdominal aortic aneurysm in Behçet's disease via mendelian randomization and integrated bioinformatics analyses
Source: J Cell Mol Med. 2024 May 24;28(10):e18398. doi: 10.1111/jcmm.18398 (PMC11117452; doi:10.1111/jcmm.18398)
Supplement: Supplementary file 1 — Appendix S1: [file JCMM-28-e18398-s001.docx]

**Supplementary Material**

**Supplementary Table S1.** Clinical characteristics of the patients.

| Characteristics | BD without AAA | BD with AAA | *P* value |
| --- | --- | --- | --- |
|  | N=8 | N=4 |  |
| Age, years | 37.63±5.37 | 41.50±4.80 | 0.252 |
| Gender, n(%) |  |  | 0.5152 |
| Male | 6(66.67) | 4(100.00) |  |
| Female | 2(33.33) | 0(0.00) |  |
| hs-CRP, mg/L | 4.00±1.52 | 5.65±2.02 | 0.1404 |
| ESR, mm/h | 7.75±2.82 | 9.75±4.65 | 0.3685 |
| Smoking, n(%) | 1(12.50) | 1(25.00) | 1 |
| DM, n(%) | 0(0) | 0(0) | 1 |
| Hyperlipidemia, n(%) | 1(12.50) | 1(25.00) | 1 |
| Hypertension, n(%) | 0(0) | 0(0) | 1 |

Abbreviations: BD, Behçet's disease; AAA, Abdominal aortic aneurysm; hs-CRP, hypersensitive C-reactive protein; ESR, Erythrocyte Sedimentation Rate; DM, Diabetes.

**Supplementary Table S2.** Primers used in the study.

| Gene | Forward primer | Reverse primer |
| --- | --- | --- |
| CD2 | AGCCTGAGTGCAAAATTCAAGT | AAAACGAGCAGTGCCACAAAG |
| CD247 | GGCACAGTTGCCGATTACAGA | CTGCTGAACTTCACTCTCAGG |
| CCR7 | TGAGGTCACGGACGATTACAT | GTAGGCCCACGAAACAAATGAT |
| β-actin | CATGTACGTTGCTATCCAGGC | CTCCTTAATGTCACGCACGAT |

**Supplementary Table S3.** The details of the IVs for BD with AAA in MR analysis.

| SNP | effect_allele  .exposure | other_allele  .exposure | effect_allele  .outcome | other_allele  .outcome | Pval .exposure | pval.outcome |
| --- | --- | --- | --- | --- | --- | --- |
| rs111388851 | G | T | G | T | 1.31E-05 | 0.870009 |
| rs116187203 | C | T | C | T | 1.73E-05 | 0.0305605 |
| rs116216407 | T | C | T | C | 8.16E-06 | 0.332112 |
| rs141560723 | A | G | A | G | 1.03E-05 | 0.170348 |
| rs1819253 | G | T | G | T | 1.42E-07 | 0.755437 |
| rs34746455 | T | G | T | G | 4.31E-05 | 0.930585 |
| rs3736054 | A | C | A | C | 3.13E-05 | 0.665475 |
| rs4353945 | C | T | C | T | 2.95E-05 | 0.363495 |
| rs5017480 | T | C | T | C | 3.93E-05 | 0.843539 |
| rs58527931 | A | G | A | G | 1.50E-05 | 0.608887 |
| rs72637670 | T | C | T | C | 1.73E-05 | 0.405324 |
| rs72831473 | A | T | A | T | 2.31E-05 | 0.28629 |
| rs72869950 | C | T | C | T | 2.99E-05 | 0.144396 |
| rs75930610 | T | G | T | G | 1.23E-05 | 0.934613 |
| rs79642439 | C | G | C | G | 4.09E-05 | 0.191743 |
| rs8042947 | G | A | G | A | 2.06E-05 | 0.543068 |

Abbreviations: MR, Mendelian randomization analysis; SNP, single nucleotide polymorphisms; BD, Behçet's disease; AAA, Abdominal aortic aneurysm.

**Supplementary Table S4.** Mendelian randomization estimates of the casual relationships between BD and AAA risks

| method | nSNP | Beta | P-Value | OR | 95% CI |
| --- | --- | --- | --- | --- | --- |
| MR Egger | 16 | 0.0652 | 0.1095 | 1.0674 | 0.9905-1.1502 |
| Weighted median | 16 | 0.0275 | 0.1923 | 1.0279 | 0.9862-1.0713 |
| Inverse variance weighted | 16 | 0.0377 | 0.0126 | 1.0384 | 1.0081-1.0696 |
| Simple mode | 16 | 0.0171 | 0.6093 | 1.0173 | 0.9539-1.0848 |
| Weighted mode | 16 | 0.0208 | 0.4757 | 1.0210 | 0.9656-1.0797 |

Abbreviations: See Supplementary Table S1.

**Supplementary Table S5.** Functional enrichment analysis of BD-related DEGs in AAA.

| Description | Count | Gene |
| --- | --- | --- |
| **Biological Process** |  |  |
| T cell activation | 17 | BCL11B/CCR6/CCR7/CD2/CD3D/CD3G/CD7/FYN/ICOS/IL7R/ITK/PIK3R1/PRKCQ/RASGRP1/SATB1/TIGIT/ZAP70 |
| T cell receptor signaling pathway | 12 | CCR7/CD247/CD3D/CD3G/FYN/ITK/PIK3R1/PRKCQ/PVRIG/SKAP1/TRAT1/ZAP70 |
| antigen receptor-mediated signaling pathway | 12 | CCR7/CD247/CD3D/CD3G/FYN/ITK/PIK3R1/PRKCQ/PVRIG/SKAP1/TRAT1/ZAP70 |
| lymphocyte activation | 17 | BCL11B/CCR6/CCR7/CD2/CD3D/CD3G/CD7/FYN/ICOS/IL7R/ITK/PIK3R1/PRKCQ/RASGRP1/SATB1/TIGIT/ZAP70 |
| immune system process | 37 | AHSP/ALAS2/BCL11B/BIRC3/BTN3A2/CCR6/CCR7/CD2/CD247/CD3D/CD3G/CD7/CD96/CRIP1/ESAM/EVL/FCGR3B/FYN/GZMA/ICOS/IL7R/ITGB7/ITK/ITLN1/KLRB1/PIK3R1/PRKCQ/PVRIG/RACGAP1/RASGRP1/RPS24/SATB1/SKAP1/TBC1D10C/TIGIT/TRAT1/ZAP70 |
| T cell differentiation in thymus | 7 | BCL11B/CCR6/CCR7/CD3D/IL7R/RASGRP1/ZAP70 |
| immune response | 28 | BIRC3/BTN3A2/CCR6/CCR7/CD247/CD3D/CD3G/CD7/CD96/CRIP1/EVL/FCGR3B/FYN/GZMA/ICOS/IL7R/ITGB7/ITK/ITLN1/KLRB1/PIK3R1/PRKCQ/PVRIG/RASGRP1/SKAP1/TBC1D10C/TRAT1/ZAP70 |
| immune response-activating cell surface receptor signaling pathway | 12 | CCR7/CD247/CD3D/CD3G/FYN/ITK/PIK3R1/PRKCQ/PVRIG/SKAP1/TRAT1/ZAP70 |
| leukocyte cell-cell adhesion | 11 | CCR7/FYN/ICOS/IL7R/ITGB7/PIK3R1/PRKCQ/RASGRP1/SKAP1/TIGIT/ZAP70 |
| positive regulation of leukocyte cell-cell adhesion | 9 | CCR7/FYN/ICOS/IL7R/PIK3R1/PRKCQ/RASGRP1/SKAP1/ZAP70 |
| **Cellular Component** |  |  |
| T cell receptor complex | 6 | CD247/CD3D/CD3G/SKAP1/TRAT1/ZAP70 |
| plasma membrane receptor complex | 7 | CD247/CD3D/CD3G/ITGB7/SKAP1/TRAT1/ZAP70 |
| immunological synapse | 4 | GZMA/PRKCQ/SKAP1/ZAP70 |
| receptor complex | 8 | CD247/CD3D/CD3G/ITGB7/ITLN1/SKAP1/TRAT1/ZAP70 |
| Set1C/COMPASS complex | 2 | USF1/WDR82 |
| membrane raft | 6 | BIRC3/CD2/FYN/ITLN1/SKAP1/ZAP70 |
| membrane microdomain | 6 | BIRC3/CD2/FYN/ITLN1/SKAP1/ZAP70 |
| membrane region | 6 | BIRC3/CD2/FYN/ITLN1/SKAP1/ZAP70 |
| perinuclear endoplasmic reticulum | 2 | FYN/PIK3R1 |
| plasma membrane protein complex | 7 | CD247/CD3D/CD3G/ITGB7/SKAP1/TRAT1/ZAP70 |
| **Molecular Function** |  |  |
| C-C chemokine receptor activity | 2 | CCR6/CCR7 |
| T cell receptor binding | 2 | CD3G/FYN |
| G protein-coupled purinergic nucleotide receptor activity | 2 | P2RY10/P2RY8 |
| protein tyrosine kinase binding | 4 | CD2/CD247/PIK3R1/TRAT1 |
| non-membrane spanning protein tyrosine kinase activity | 3 | FYN/ITK/ZAP70 |
| G protein-coupled nucleotide receptor activity | 2 | P2RY10/P2RY8 |
| C-C chemokine binding | 2 | CCR6/CCR7 |
| transmembrane receptor protein tyrosine kinase adaptor activity | 2 | PIK3R1/TRAT1 |
| protein kinase binding | 9 | BCL2L1/CD2/CD247/PIK3R1/RACGAP1/RPS18/SKAP1/TRAT1/USF1 |
| purinergic nucleotide receptor activity | 2 | P2RY10/P2RY8 |
| **KEGG** |  |  |
| T cell receptor signaling pathway | 10 | CD247/CD3D/CD3G/FYN/ICOS/ITK/PIK3R1/PRKCQ/RASGRP1/ZAP70 |
| PD-L1 expression and PD-1 checkpoint pathway in cancer | 7 | CD247/CD3D/CD3G/PIK3R1/PRKCQ/RASGRP1/ZAP70 |
| Primary immunodeficiency | 4 | CD3D/ICOS/IL7R/ZAP70 |
| Th1 and Th2 cell differentiation | 5 | CD247/CD3D/CD3G/PRKCQ/ZAP70 |
| Hematopoietic cell lineage | 5 | CD2/CD3D/CD3G/CD7/IL7R |
| Th17 cell differentiation | 5 | CD247/CD3D/CD3G/PRKCQ/ZAP70 |
| Natural killer cell mediated cytotoxicity | 5 | CD247/FCGR3B/FYN/PIK3R1/ZAP70 |
| Cell adhesion molecules (CAMs) | 5 | CD2/ESAM/ICOS/ITGB7/TIGIT |
| NF-kappa B signaling pathway | 4 | BCL2L1/BIRC3/PRKCQ/ZAP70 |
| Chagas disease (American trypanosomiasis) | 4 | CD247/CD3D/CD3G/PIK3R1 |

# Abbreviations: BD, Behçet's disease; AAA, Abdominal aortic aneurysm; DEG, differentially expressed genes; KEGG, kyoto encyclopedia of genes and genomes.

**Supplementary Table S6.** Complete list of DEGs from three algorithms via CytoHubba plug-in.

| Degree | Betweenness | Closeness | Intersection |
| --- | --- | --- | --- |
| ZAP70 | CD3G | ZAP70 | CD3G |
| CD3D | ALAS2 | CD3D | IL7R |
| CD2 | IL7R | CD247 | CD247 |
| CD247 | BCL2L1 | CD2 | CD2 |
| IL7R | CD247 | CD3G | ZAP70 |
| ITK | CD2 | IL7R | CCR6 |
| CD3G | ZAP70 | ITK | ITK |
| GZMA | CCR6 | GZMA | TRAT1 |
| GZMK | ITK | GZMK | CD3D |
| CCR7 | TRAT1 | ICOS | TRAF3IP3 |
| ICOS | TMOD1 | CCR7 | CCR7 |
| KLRB1 | CD3D | CD96 | TBC1D10C |
| CD96 | TRAF3IP3 | KLRB1 | GZMA |
| TIGIT | HBD | TIGIT | GZMK |
| FCGR3B | CCR7 | TRAT1 | BCL11B |
| CD7 | SLC25A39 | CD7 | ICOS |
| TRAT1 | TBC1D10C | FCGR3B | TIGIT |
| FYN | GZMA | FYN | CD96 |
| TRAF3IP3 | GZMK | CCR6 | KLRB1 |
| PTPRCAP | BCL11B | TRAF3IP3 | PTPRCAP |
| CCR6 | ICOS | PTPRCAP |  |
| PVRIG | BACH2 | BCL11B |  |
| SKAP1 | RPL9 | PIK3R1 |  |
| BCL11B | TIGIT | SKAP1 |  |
| IL10RA | CD96 | PVRIG |  |
| PIK3R1 | KLRB1 | IL10RA |  |
| ITGB7 | AHSP | PRKCQ |  |
| PRKCQ | HBQ1 | ITGB7 |  |
| TBC1D10C | PTPRCAP | TBC1D10C |  |
| RASGRP1 | BIRC3 | BCL2L1 |  |

Abbreviations: DEG, differentially expressed genes.

**Supplementary Table S7 Therapeutic agents screening**

| Term | P-value | Genes |
| --- | --- | --- |
| methotrexate CTD 00006299 | 0.002810145 | CD2, CD247 |
| Cadmium dichloride BOSS | 0.003296484 | CD2 |
| alpha-D-Mannose TTD 00001913 | 0.004044678 | CD2 |
| Vitinoin CTD 00007069 | 0.004439114 | CD2, CD247 |
| Tributyltin CTD 00000610 | 0.00524101 | CCR7 |
| Ammonium hexachloroplatinate(IV) CTD 00000945 | 0.005838817 | CCR7 |
| IVERMECTIN CTD 00006182 | 0.005988231 | CCR7 |
| Bandrowski's base CTD 00002216 | 0.005988231 | CCR7 |
| FERROUS SULFATE CTD 00001009 | 0.008525987 | CCR7 |
| TACROLIMUS MONOHYDRATE CTD 00007118 | 0.008675133 | CD2 |
| albendazole CTD 00007095 | 0.008973378 | CCR7 |
| MERCURY BOSS | 0.009867756 | CD2 |
| FITC BOSS | 0.010016767 | CCR7 |
| Sodium dodecyl sulfate CTD 00006753 | 0.010761596 | CCR7 |
| Edetate sodium BOSS | 0.012845131 | CD2 |
| 15-Acetyldeoxynivalenol CTD 00002073 | 0.013291222 | CCR7 |
| TITANIUM BOSS | 0.014925739 | CD2 |
| methyprylon BOSS | 0.018930083 | CD2 |
| Dinoprostone CTD 00007049 | 0.018930083 | CCR7 |
| Oxazolone CTD 00006449 | 0.019374339 | CCR7 |
| Rifampicin CTD 00006701 | 0.019818461 | CCR7 |
| CADMIUM BOSS | 0.0212979 | CD2 |
| eugenol CTD 00005949 | 0.023957137 | CCR7 |
| histamine BOSS | 0.02675888 | CD2 |
| bilirubin BOSS | 0.027053488 | CD2 |
| COPPER BOSS | 0.027348037 | CD2 |
| formic acid BOSS | 0.027642526 | CD2 |
| sodium chloride BOSS | 0.027789749 | CD2 |
| AGN-PC-0JHFVD BOSS | 0.028378489 | CD2 |
| Dinoprostone BOSS | 0.029114081 | CCR7 |
| cyclophosphamide BOSS | 0.029408213 | CD2 |
| Alitretinoin CTD 00003402 | 0.031465479 | CCR7 |
| 1-NITROPYRENE CTD 00001569 | 0.034692448 | CCR7 |
| 1-chloro-2,4-dinitrobenzene CTD 00005848 | 0.035864112 | CCR7 |
| clindamycin HL60 UP | 0.048980071 | CCR7 |


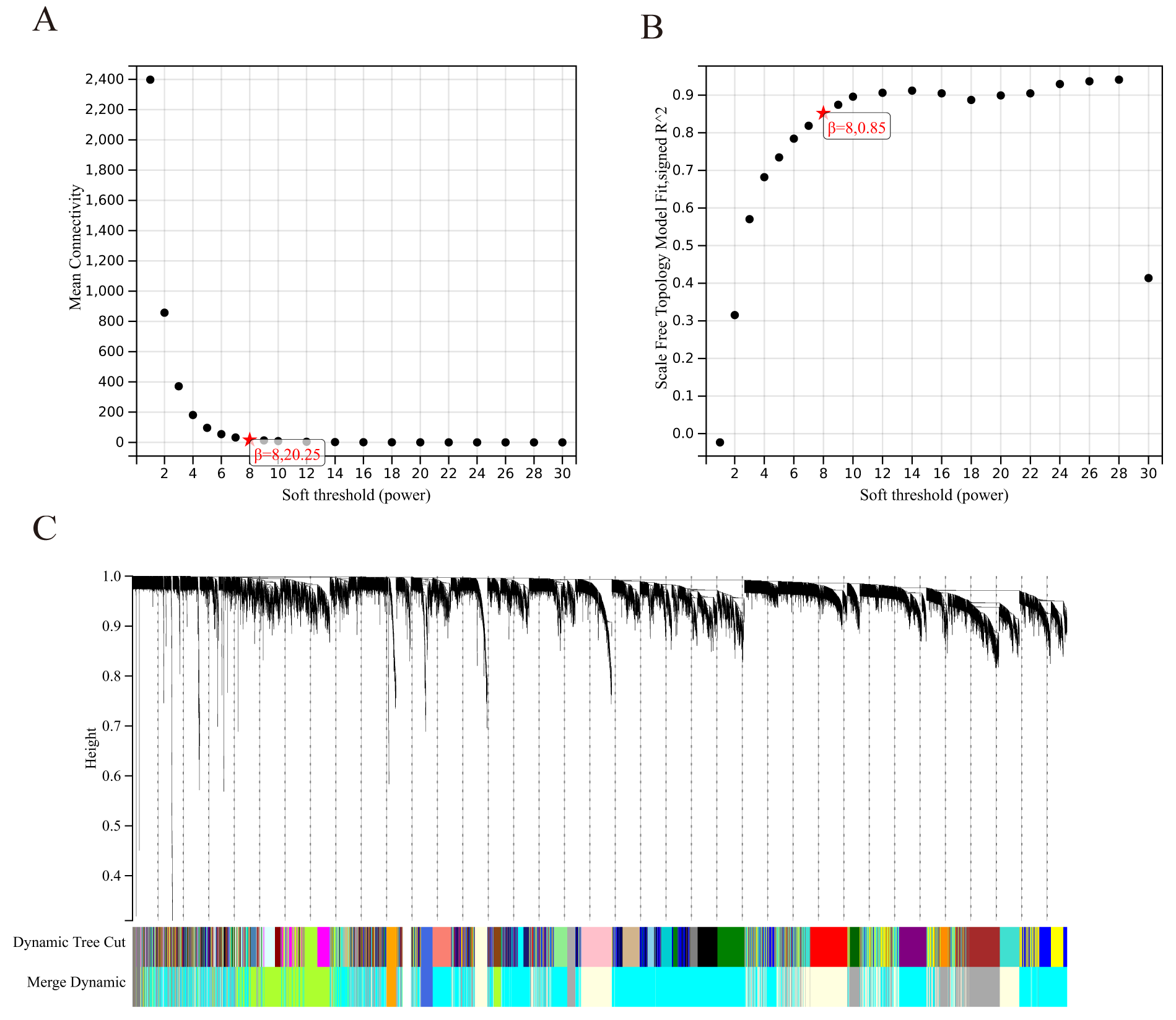


**Supplementary Figure S1.** Soft thereshold selection and gene cluster tree via WGCNA of BD**.**

(A-B) The soft threshold selection. β=8 was choose as the most appropriate threshold.

(C) Gene cluster tree of different modules.

Abbreviations: WGCNA, weighted gene co-expression network analysis; BD, Behçet's disease.


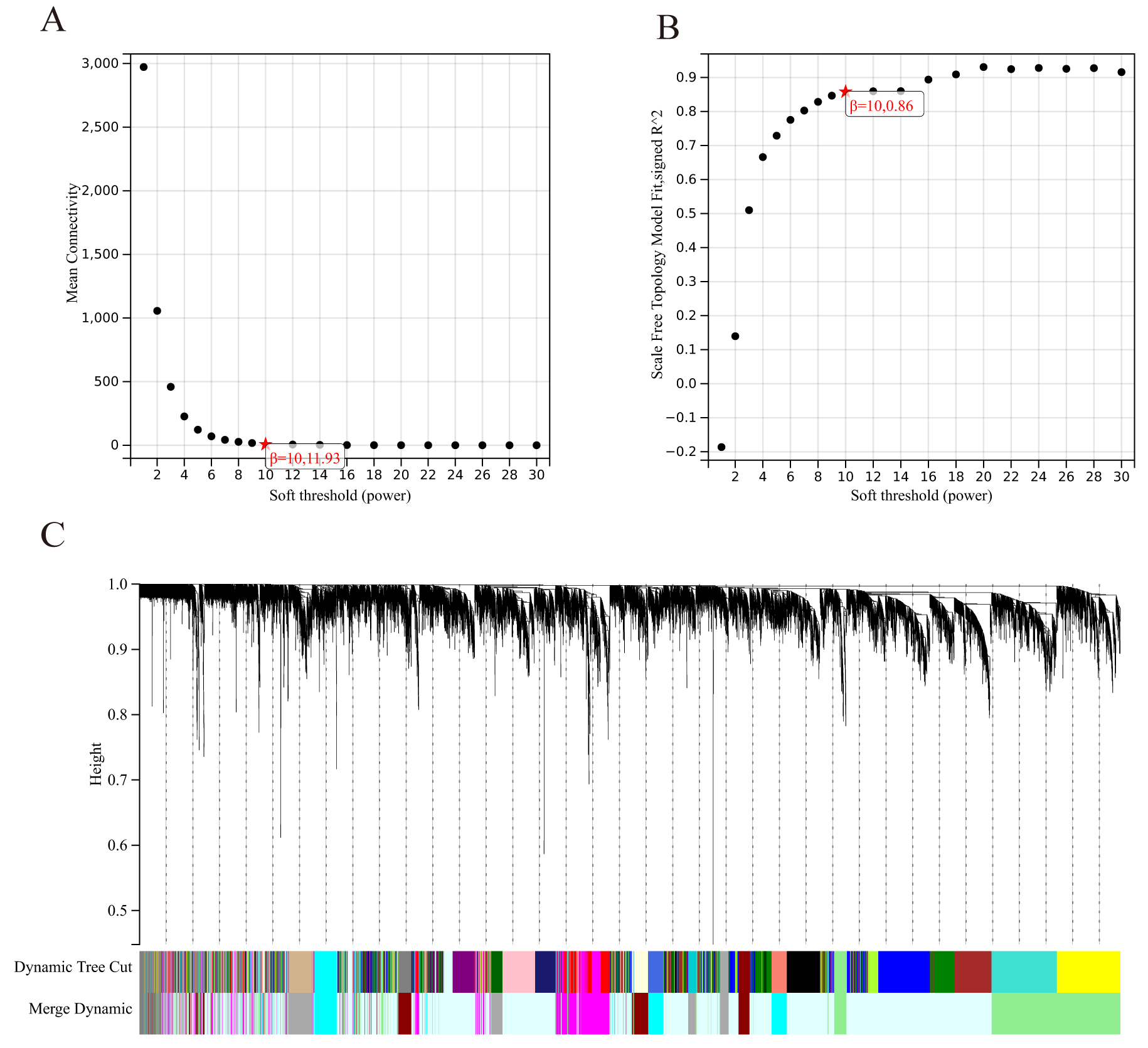


**Supplementary Figure S2.** Soft thereshold selection and gene cluster tree via WGCNA of AAA.

(A-B) soft threshold selection. β=10 was chosen as the most appropriate threshold.

(C) Gene cluster tree of different modules.

Abbreviations: WGCNA, weighted gene co-expression network analysis; AAA, Abdominal aortic aneurysm.


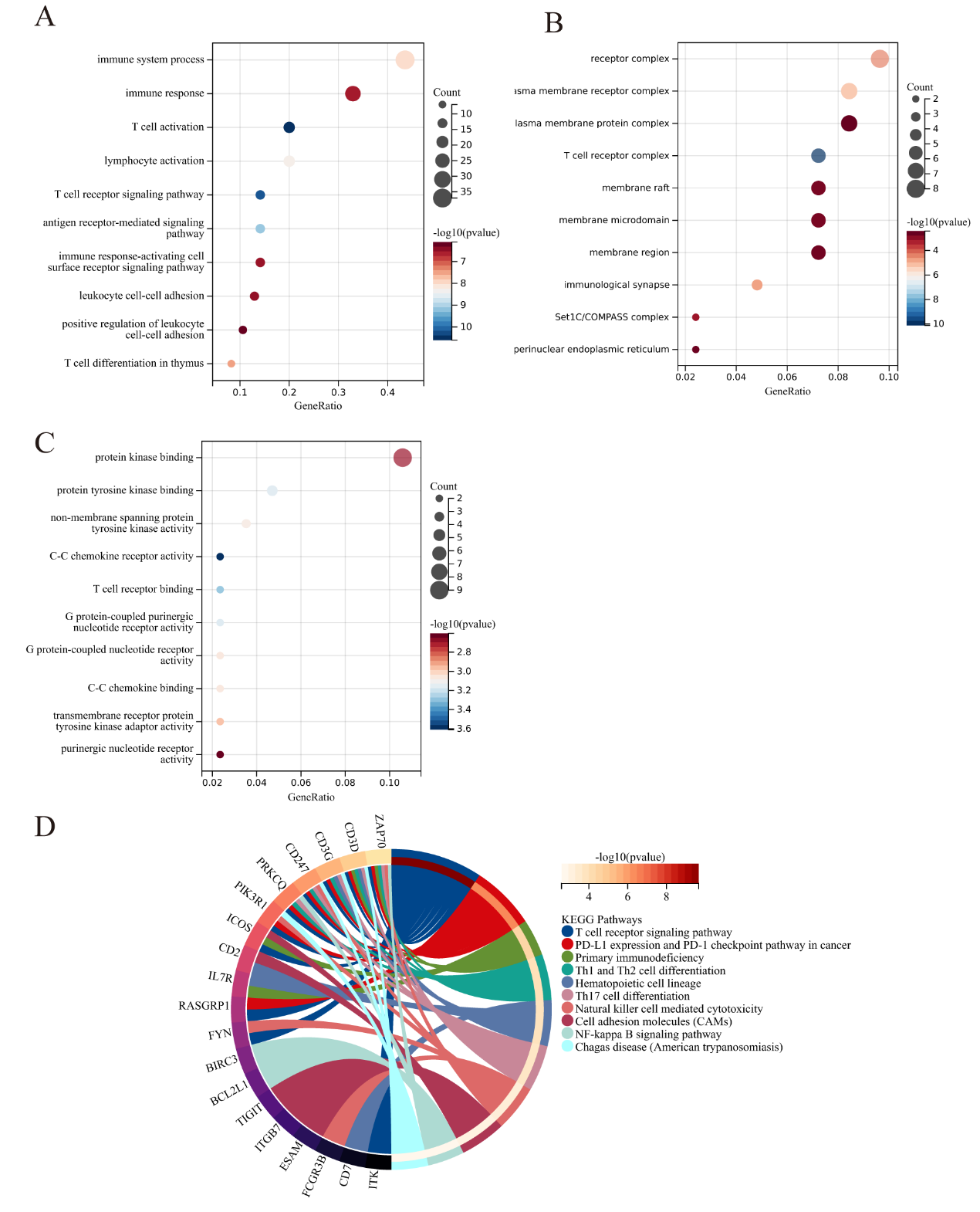


**Supplementary Figure S3**. Functional enrichment analysis of BD-related DEGs in AAA.

(A-C) GO analysis (BP, CC, and MF) of BD-related DEGs in AAA. The size of the circles corresponds to the number of genes, and the color indicates the level of significance.

(D) KEGG pathway analysis of BD-related DEGs in AAA.


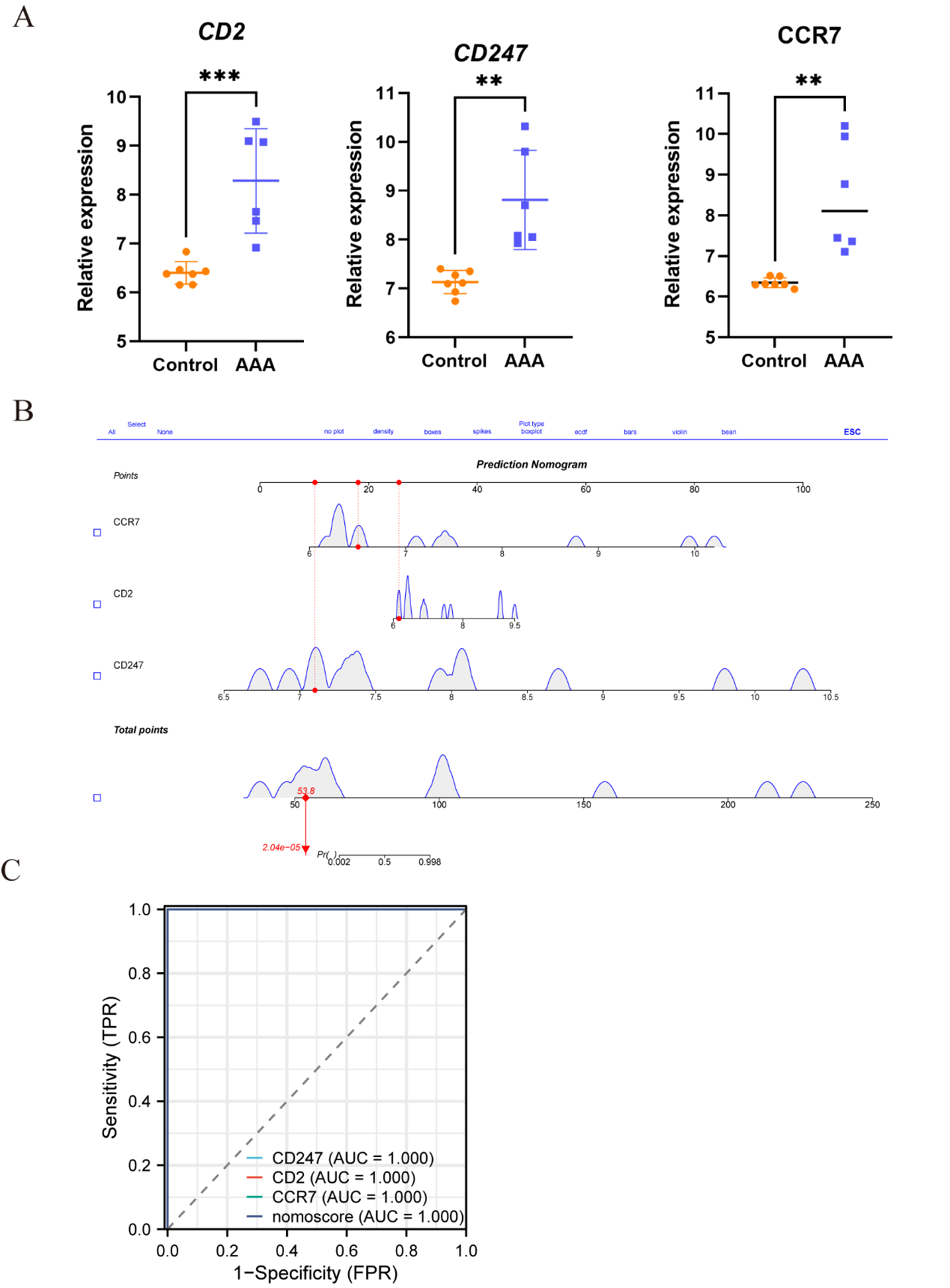


**Supplementary Figure S4.** The diagnostic value of candidate hub biomarkers and nomogram construction.

(A) Differences in expression between AAA and control groups for the three genes. **, P < 0.01; ***, P < 0.001.

(B) The diagnostic efficacy of the three genes in identifying AAA with BD was evaluated using the ROC curve, with each panel presenting the AUC and its corresponding 95% CI.

(C) Utilizing the three genes, a nomogram was constructed to aid in the diagnosis of AAA with BD.

(D) The diagnostic performance of the nomogram in AAA with BD from the ROC curve.

Abbreviations: BD, Behçet's disease; AAA, Abdominal aortic aneurysm; ROC, receiver operating characteristic curve. AUC, area under the curve; CI, confidence interval.


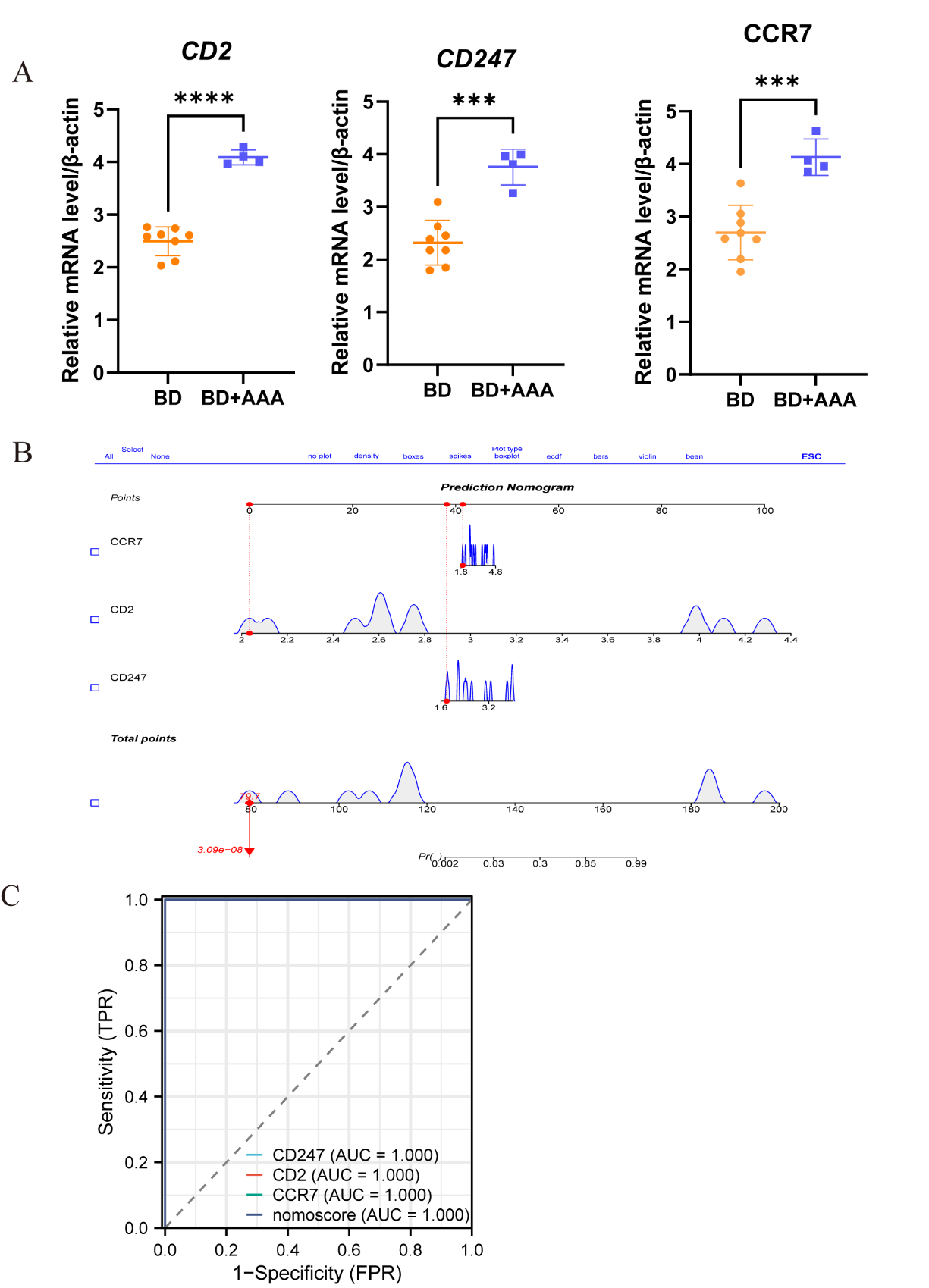


**Supplementary Figure S5.** Validation of expression of three hub genes by RT-qPCR and nomogram construction.

(A) Expression of CD2, CD247 and CCR7 between samples from BD complicated with AAA and the BD groups. ****p* < 0.001; ****, *p* < 0.0001.

(B) A nomogram was developed using three externally validated DEGs. Each DEG corresponds to a score on the nomogram. The final score was determined by adding the scores for each DEG.

(C) ROC curve of the nomogram revealed DEGs with prominent predictive value for AAA in BD patients.


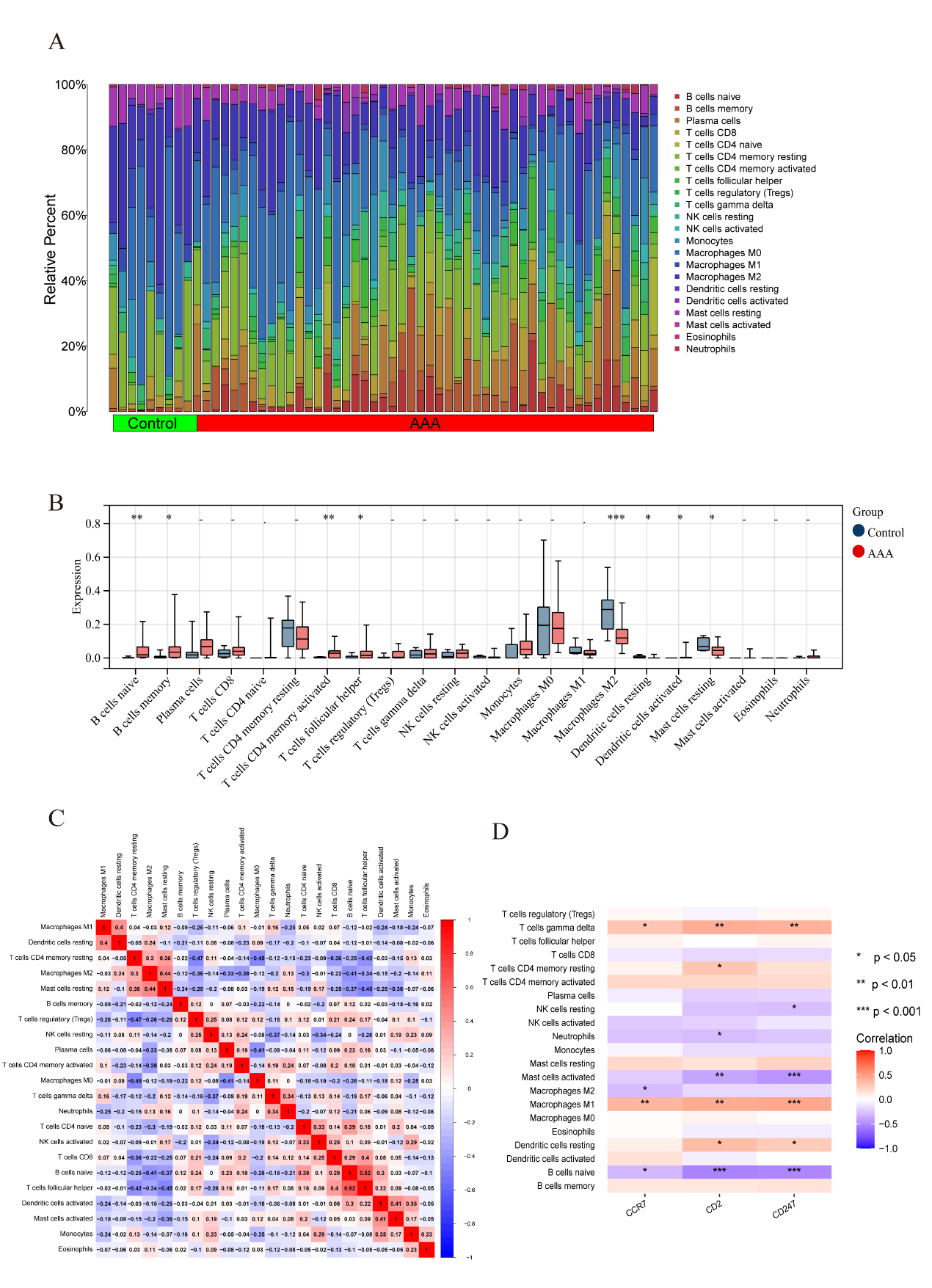


**Supplementary Figure S6**. Immunological alterations between control and AAA groups, and correlations between hub DGEs and immunological features in AAA.

(A) Bar plot illustrating the distribution of immune cells in different samples.

(B) Boxplot comparing the expression of immune cells between AAA and control groups. *p < 0.05, **p < 0.01, ***p < 0.001.

(C) Heatmap representing the correlations among different immune cells implicated in the pathogenesis of AAA.

(D) Correlation analysis assessing the relationship between immune cell infiltrations and the three identified hub DGEs.


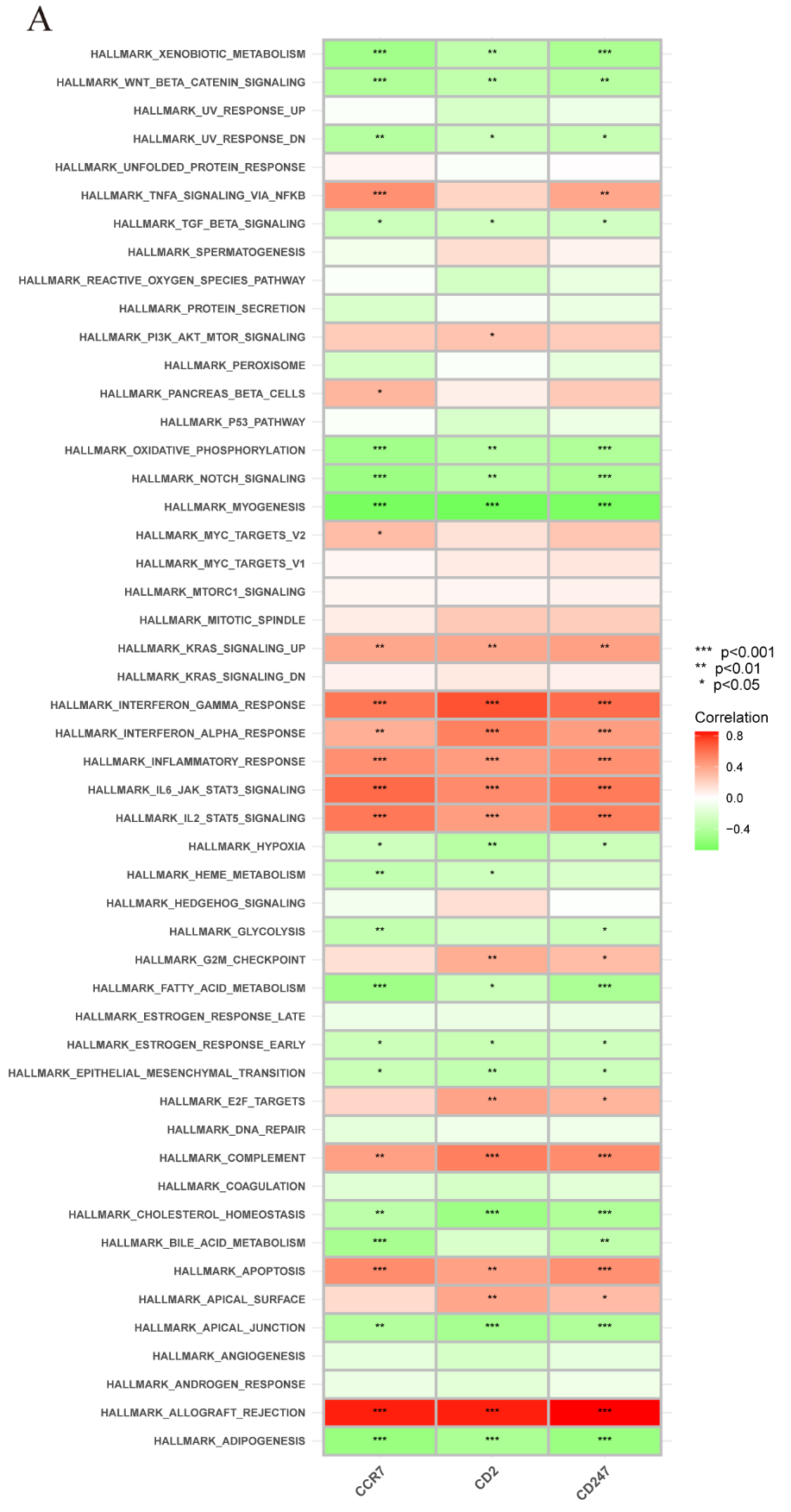


**Supplementary Figure S7.** Correlation analysis of the three candidate biomarkers with hallmark gene sets via ssGSEA

Abbreviations: ssGSEA, Single-sample gene-set enrichment analysis.


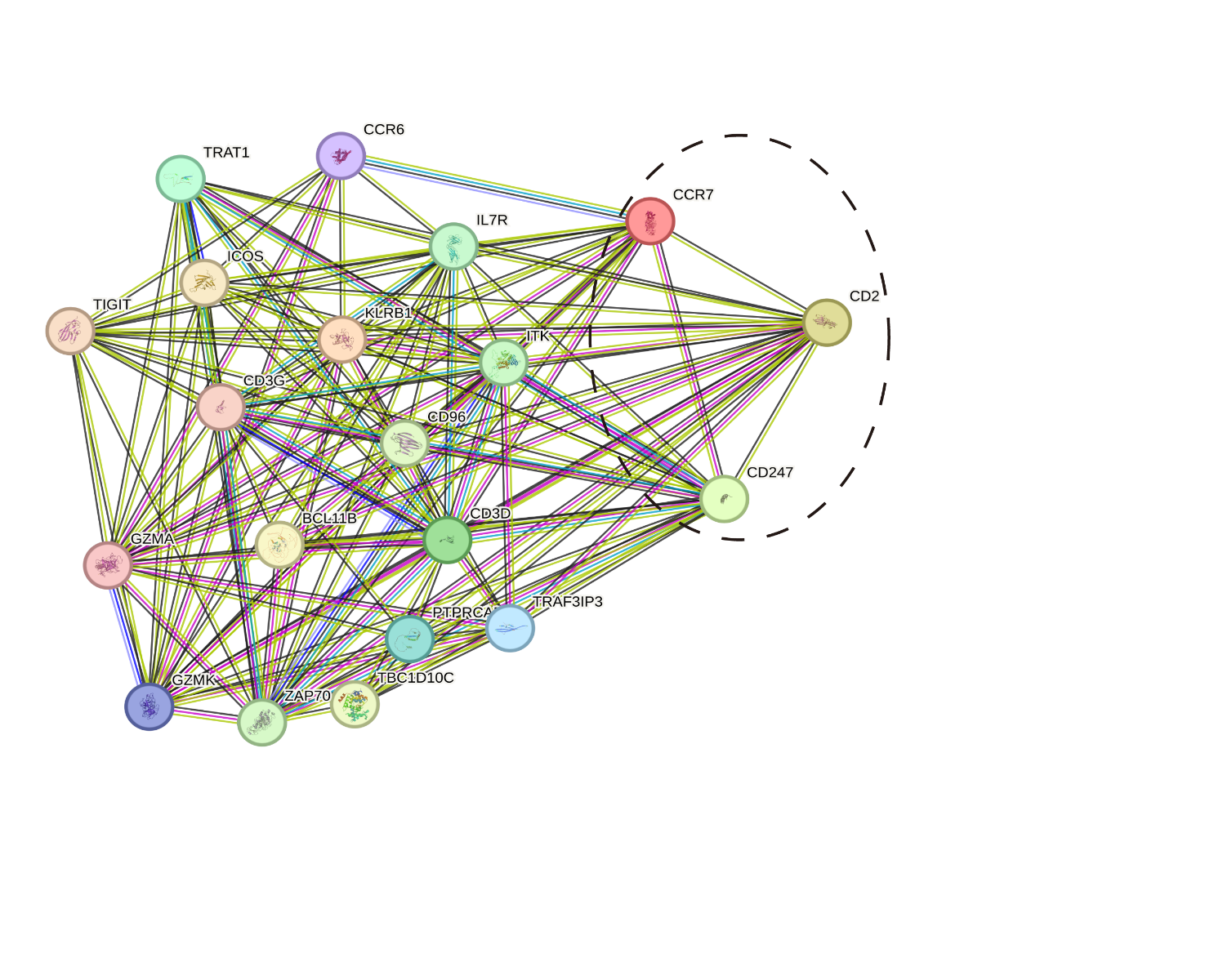


**Supplementary Figure S8.** The interaction among three identified genes in diagnosing AAA with BD.
